# Recent Progress in Multiphase Thermoelectric Materials

**DOI:** 10.3390/ma14206059

**Published:** 2021-10-14

**Authors:** Raphael Fortulan, Sima Aminorroaya Yamini

**Affiliations:** 1 Materials and Engineering Research Institute, Sheffield Hallam University, Sheffield S1 1 WB, UK; b9044884@my.shu.ac.uk; 2 Department of Engineering and Mathematics, Sheffield Hallam University, Sheffield S1 1 WB, UK

**Keywords:** thermoelectric materials, multiphase, composite, energy filtering, magnetic effect, phonon scattering

## Abstract

Thermoelectric materials, which directly convert thermal energy to electricity and vice versa, are considered a viable source of renewable energy. However, the enhancement of conversion efficiency in these materials is very challenging. Recently, multiphase thermoelectric materials have presented themselves as the most promising materials to achieve higher thermoelectric efficiencies than single-phase compounds. These materials provide higher degrees of freedom to design new compounds and adopt new approaches to enhance the electronic transport properties of thermoelectric materials. Here, we have summarised the current developments in multiphase thermoelectric materials, exploiting the beneficial effects of secondary phases, and reviewed the principal mechanisms explaining the enhanced conversion efficiency in these materials. This includes energy filtering, modulation doping, phonon scattering, and magnetic effects. This work assists researchers to design new high-performance thermoelectric materials by providing common concepts.

## 1. Introduction

Thermoelectric (TE) materials have been attracting a great deal of interest because of their applications in energy recovery from industrial waste heat and high-efficiency cooling of next-generation integrated circuits [1]. The efficiency of TE materials is characterised by a dimensionless figure of merit $zT=S^{2}\sigma T/\left(\kappa_{e}+\kappa_{l}\right)$, where T, S, σ, $\kappa_{e}$ and $\kappa_{l}$ are the absolute temperature, Seebeck coefficient, electrical conductivity, and electronic and lattice components of the total thermal conductivity ($\kappa_{t}$), respectively. Most thermoelectric materials used in commercial applications have a *z*T of around 1 [2], which corresponds to an efficiency of roughly 10% in the medium temperature range [3]. To increase *z*T, one would need to increase S and σ and decrease $\kappa_{t}$. These parameters are interrelated though; the electronic thermal conductivity increases with the increase of electrical conductivity, and S and σ are inversely proportional [4]. Therefore, optimising these parameters is very challenging and the key to achieving higher efficiency.

One of the most successful approaches to improve the figure of merit is reducing the lattice thermal conductivity, and over the years, various phonon engineering approaches have been used to enhance phonon scattering and decrease $\kappa_{l}$ by taking advantage of nanoprecipitates [5,6,7], alloying elements [8,9,10], nanostructured grain boundaries [11,12,13,14], ionised impurities [15,16], and superlattices [17].

A series of band structure engineering approaches have also been employed to improve the electronic properties [18,19,20]. Strategies such as quantum confinement [21,22], modulation doping [23,24,25], introducing resonance energy to the electronic density of states [26,27], and energy filtering [28] are being actively pursued. These strategies are adopted to modify the band structure and transport properties of the thermoelectric materials by either tuning the electrical conductivity and the Seebeck coefficient independently or by increasing them simultaneously. In practice, the best results were achieved with a significant increase in one of these parameters and a slight decrease in the other, resulting in an increase in the power factor ($S^{2}\sigma )$ and *z*T.

**Figure 1:**
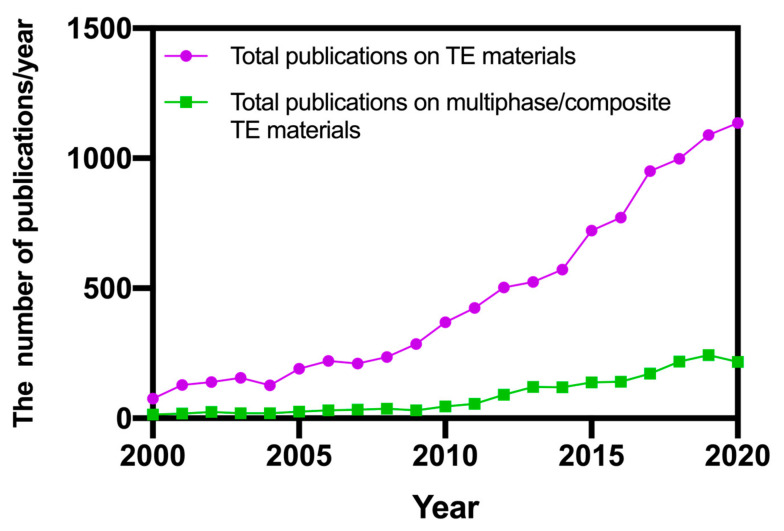
Yearly number of publications on thermoelectric materials, compared with publications on multiphase/composite thermoelectric materials.

The development of highly efficient thermoelectric materials encountered a bottleneck when the exploration of single-phase alloys was exhausted. Therefore, investigating multiphase compounds is the most viable strategy to enhance the thermoelectric performance of bulk materials, where higher degrees of freedom are available to design new materials and tune their electronic transport properties [29,30,31]. Figure 1 presents the number of yearly publications on thermoelectric materials, indexed by the Scopus database, compared with the numbers of papers published on multiphase/composite thermoelectric materials, indicating an increasing interest of the research community on this topic over the last five years.

The combination of several phases can improve the electrical conductivity, Seebeck coefficient, and thermal conductivity [32]. Here, we summarise the main strategies discovered to date to increase the thermoelectric efficiency in multiphase materials. This includes: (1) energy filtering, creating potential barriers in the electronic band structure of the main phase through interfaces with the secondary phases. This results in an increase in the overall Seebeck coefficient [33,34,35]; (2) modulation doping, where the heterojunctions between secondary phases with larger bandgaps and higher carrier concentrations than the matrix are used to greatly increase the electrical conductivity of the multiphase compounds [36,37,38]; (3) phonon scattering by interfaces, grain boundaries, and defects to reduce the lattice thermal conductivity [39,40,41,42]; and (4) magnetic effects, which utilises the magnon-drag mechanism in magnetic materials [43,44,45], semiconductors doped with magnetic elements [46,47,48], or semiconductors containing secondary magnetic phases [49,50] to improve the thermoelectric efficiency [51,52].

## 2. Energy Filtering

The concept of energy filtering in thermoelectric materials was first introduced and studied in the latter half of the last century [53]. The research on this field has been renewed and developed since the 1990s. In general, the Seebeck coefficient increases with an increase of the barrier height [54,55] while the electrical conductivity decreases [56,57,58]. In the presence of multiple potential barriers, the bipolar effect can be suppressed, decreasing the flow of minority charge carriers and reducing the decrease in the electrical conductivity [59,60,61,62].

Conventionally, the energy filtering effect could be understood by solving the Boltzmann transport equation (BTE) using the relaxation time approximation [63,64]. The BTE equation expresses all thermoelectric transport coefficients as a function of the energy-dependent relaxation times of the charge carriers $\tau \left(E\right)$ and the Fermi level $E_{F}$. The energy filtering effect can be readily verified considering the Seebeck coefficient expression [65]:

(1) $$S=-\frac{1}{qT}\frac{\int \left(E-E_{F}\right)\sigma^{\prime }\left(E\right)dE}{\int \sigma^{\prime }\left(E\right)dE}$$

where T is the absolute temperature, E is the charge carrier energy, $E_{F}\;$ is the Fermi level, and $\sigma^{\prime }$ is the differential conductivity. The Seebeck coefficient, as shown in Equation (1), is the energy average weighted by the electrical conductivity:

(2) $$S=-\frac{1}{qT}\langle E-E_{F}\rangle {}_{\sigma^{\prime }}$$

implying that an asymmetry between the density of states and the Fermi level can create higher Seebeck coefficients.

**Figure 2:**
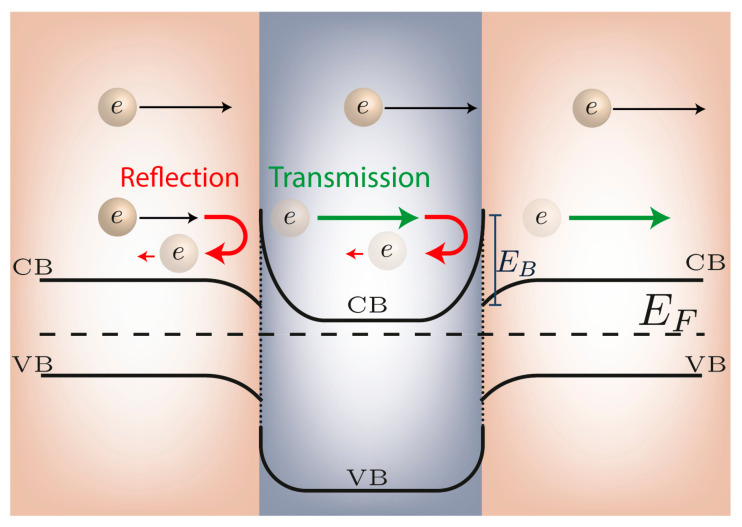
Energy filtering effect, showing that lower energy electrons are scattered by a potential barrier.

The implementation of energy barriers in thermoelectric materials is made in the form of either nanoparticles or grain boundary interfaces embedded in the bulk host matrix [66]. At these interfaces, the carriers with higher energy will pass the interface preferentially, while the carriers with lower energy are filtered out. A high density of the interfaces ensures the positive carrier filtering effect [67]. The band bending between the two materials creates an energy barrier that reflects the charge carrier [68,69]. Figure 2 illustrates the energy filtering effect: given the partial reflection of the lower energy electron waves, the high energy electrons mostly contribute to the Seebeck coefficient.

Multiphase materials present themselves as viable candidates to take advantage of energy filtering. Given the possibilities of tuning the electronic band structure of each phase, the band bending can noticeably enhance the energy filtering effect [70,71,72,73,74,75,76,77]. In the following sections, the energy filtering effect will be discussed in multiphase materials. A Schottky or ohmic barrier will appear at the interface with a metallic secondary phase, while a heterojunction barrier will present at the interface of thermoelectric material with a semiconducting secondary phase.

### 2.1. Energy Filtering by Metal Secondary Phases

For metallic phases, the barrier height will be proportional to the work function of both the semiconductor and the metal and the electron affinity of the semiconductor. The work function, Φ, is defined as the minimum energy required to remove an electron from the surface of a material, and its value is equal to the energy difference between the vacuum and the Fermi level [78]. The electron affinity, χ, is the energy difference between the vacuum and the bottom of the conduction band. At the metal–semiconductor junction, the Schottky-Mott rule [79] defines the barrier height, $E_{B}$, to be the difference between the metals’ work function, $\Phi_{M}$, and the semiconductor electron affinity, $\chi_{S}$:

(3) $$E_{B}=\Phi_{M}-\chi_{s}$$

Small differences cause few restrictions on the movement of the electrons, and this barrier is known as ohmic. Both the work function and electron affinity depend on the surface impurity and the crystallographic orientation.

**Figure 3:**
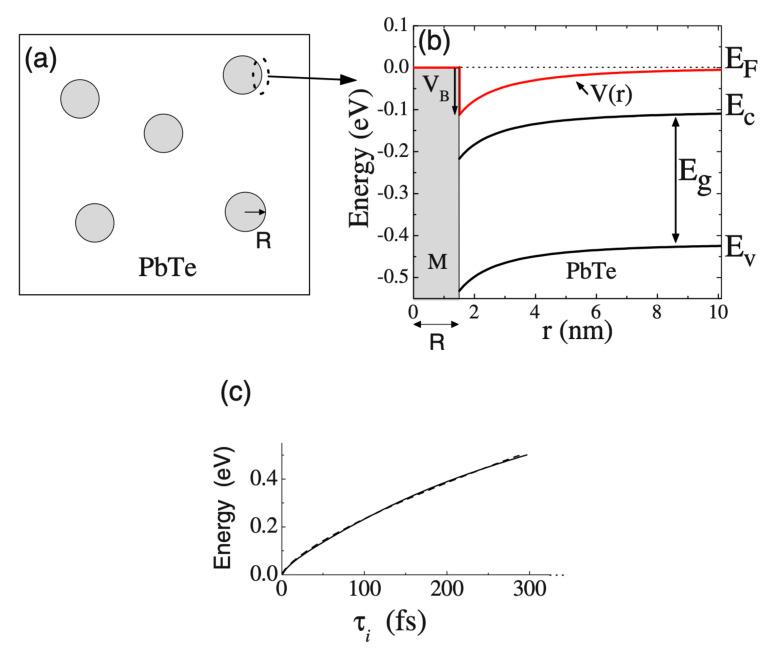
(**a**) Schematic of randomly distributed metallic Pb secondary phase in a PbTe matrix (**b**) Calculated potential V(r) and energy diagram for PbTe at 300 K, carrier concentration of 2.5 × 10^19^ cm^−3^, barrier height of 0.11 eV, and radius of 1.5 nm, where $E_{F}$ is the Fermi level, $E_{C}$ is the energy at the bottom of the conduction band, $E_{g}$ is the band gap, and $E_{V}$ is the energy at the top of the valence band (**c**) Carriers’ relaxation time as a function of their energy. Reprinted with permission from ref. [66]. Copyright 2008 Copyright American Physical Society.

Theoretically, the presence of metals can strongly affect the carrier relaxation time and consequently affects both the electrical conductivity and the Seebeck coefficient [54,80,81]. The inclusion of metallic nanoparticles was shown to enhance the Seebeck coefficient mathematically [66]. The interface of *n*-type PbTe with metallic Pb nanoparticles, with a low work function, effectively scattered the electrons and increased both the Seebeck coefficient and electrical resistivity. This mechanism is illustrated schematically in Figure 3, where spherical metallic nanoparticles are randomly distributed in a host semiconductor.

Experimental results have proved this concept [82,83,84]: for instance, Pb precipitates in the matrix of *n*-type PbTe, intrinsically doped with excess Pb, increased the Seebeck coefficient and the average resistivity from −98 μV/K and 1.2 mΩ cm for Pb_1.03_Te to −130 μV/K, and 3.2 mΩ cm for Pb_1.06_Te at 300 K, respectively [82]. The addition of metallic Sn and Cr to Cu_2_O doubled the Seebeck coefficient from 700 μV/K to 1400 μV/K [84]. Platinum nanocrystals created energy barriers in *p*-type Sb_2_T_3_ thin films [83] and caused a large reduction in carrier mobility in about 2.5 orders of magnitude due to the additional scattering of charge carriers compared to Sb_2_Te_3_ films without Pt nanocrystals. The carrier concentration increased, possibly due to the overlapping of energy bands [66]. The band overlapping increases the distance between the Fermi energy level and the valence band maxima, thereby increasing the total concentration of holes in the semiconductor matrix.

Silver nanoparticles enhanced the Seebeck coefficient of CdO-Ag composites [85]—a potential barrier of $E_{B}$ = 0.1 eV between the work function of Ag and the electron affinity of CdO increased the resistivity from 1.5 mΩ cm to 1.7 mΩ cm for a sample with 0.03% of Ag and increased the Seebeck coefficient from −120 μV/K for the pristine sample to −129 μV/K for the sample with 0.03% of Ag at 800 K.

### 2.2. Energy Filtering by Semiconducting Secondary Phases

For a semiconductor secondary phase, the difference between the bandgaps and Fermi levels of the two phases gives rise to a potential barrier at their junction [86]. The barrier height will be proportional to the difference between the electron affinity of the two semiconductors ($\chi_{1},\;\chi_{2})$. Anderson’s rule [78] allows a simple estimate of the barrier height at the conduction, $E_{BC}$, and valence, $E_{BV}$, bands:

(4) $$E_{BC}=\chi_{2}-\chi_{1}$$

(5) $$E_{BV}=\left(E_{g1}-E_{g2}\right)-E_{BC}$$

and it has been employed as a rough estimation to design multiphase materials. The actual curvature of the band bending can be found using Poisson’s equation for the electric potential [87]:

(6) $$-\nabla^{2}V=\frac{\rho }{\varepsilon }=\frac{q\left(h-n+D\right)}{\varepsilon }$$

where ε is the permittivity of the material, h and n are the holes and electrons densities, respectively, and D is the concentration of ionised impurities (extrinsic dopant). The relation of the barrier height and conductivity is suggested as [88]:

(7) $$\sigma \propto T^{-\frac{1}{2}}e^{-\frac{E_{B}}{k_{B}T}}$$

It should be noted that this equation was developed for homojunctions [89,90]. However, experimental data for heterojunctions fit this equation surprisingly well [91]. Experimental results show that energy filtering caused by dissimilar semiconducting phases can improve the power factor [92,93,94,95]. Silicon oxide particles in the (Bi_2_Te_3_)_0.2_(Sb_2_Te_3_)_0.8_ bulk alloy increased the Seebeck coefficient from 182 μV/K for the pristine sample to 218 μV/K for the sample with 1.1% volume of SiO_2_ at room temperature [92]. Yttrium oxide (Y_2_O_3_) particles embedded in a Bi_0.5_Sb_1.5_Te_3_ matrix increased the Seebeck coefficient significantly [96], deviated considerably from the ideal Pisarenko relation. The Seebeck coefficient is inversely proportional to the carrier concentration, n, by a rate of $n^{-2/3}$ for degenerate semiconductors, according to the Pisarenko relation [97]. The deviation from this ideal relationship has been used as an indication of changes in the electronic band structure of the material [98].

Randomly dispersed titanium dioxide nanoparticles (ranging from 10 to 25 nm) in a Ba_0.22_Co_4_Sb_12_ matrix increased the Seebeck coefficient [99]. Although the bandgap for Ba_0.22_Co_4_Sb_12_ was unknown, given the large bandgap of TiO_2_ (3.2 eV [100]), some influence of the energy filtering was assumed. At 300 K, the electrical conductivity decreased from 2.9 × 10^5^ S/K to 2.8 × 10^5^ S/K, and the Seebeck coefficient increased from −105 μV/K for the pristine sample to −110 μV/K for the sample with 0.8% volume of TiO_2_. The TiO_2_ particles in Bi_2_Se_3_ based materials increased the overall power factor of the composite from 0.75 × 10^−3^ W/m K to 1.07 × 10^−3^ W/m K for the sample with 10 wt.% of titanium dioxide [101]. The charge carrier concentration varied greatly with the concentration of TiO_2_, possibly due to the formation of Ti^2+^ ions during hot pressing under the vacuum.

**Figure 4:**
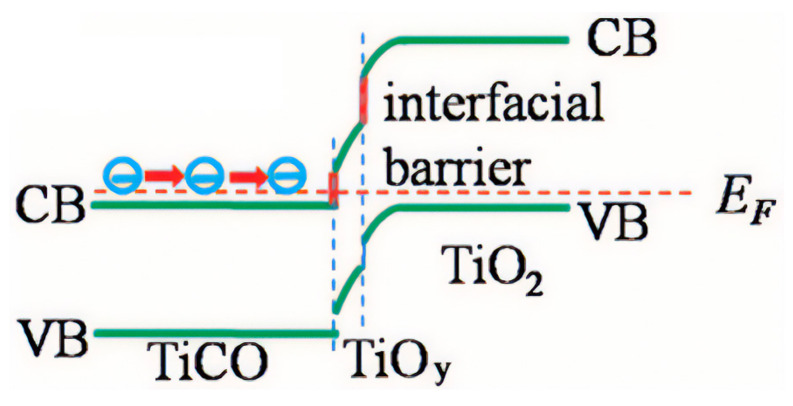
Band diagrams of the TiC_1−x_O_x_@TiO_y_-TiO_2_ heterostructured interface, where CB is the conduction band, VB is the valence band, and $E_{F}$ is the Fermi level. Reprinted from [102].

Interestingly, a double-filtering effect has been reported for a deposited TiO_2_ on TiC_1-*x*_O*_x_*@TiO*_y_* (*x* < 1, 1 < *y* < 2) heterostructures (Figure 4) [102]. TiC_1−*x*_O*_x_*, with a narrow bandgap and high electrical conductivity, in combination with TiO*_y_* and TiO_2_ nanoparticles, with wide bandgaps, produced an effective barrier height for energy filtering. The Seebeck coefficient and resistivity of the samples increased where a larger amount of TiO_2_ was deposited. The maximum value obtained for the Seebeck coefficient at 973 K was −156 μV/K, with an electrical conductivity of ~4 × 10^4^ S/m.

Silicon-based materials, although not common in thermoelectricity, have also been shown to benefit from energy filtering [103,104,105,106,107,108,109,110]. For instance, heavily doped Si with B with nanoparticles of Si has shown an increased Seebeck coefficient and electrical conductivity in a particular range of dopant concentrations [111]. The increased Fermi level for the bulk material explains the increase of the electrical conductivity, and the energy filtering effect justifies the increased Seebeck coefficient.

A half-Heusler compound of (Hf_0.6_Zr_0.4_)NiSn_0.99_Sb_0.01_, with added nanoparticles of tungsten (W), showed a maximum *z*T of 1.4 at 873 K and average *z*T of 0.9 in the temperature range of 300–973 K for the alloy with 5 wt.% tungsten nanoparticles [112].

Table 1 summarises the compositions and fabrication methods of recent studies that reported energy filtering effects in multiphase thermoelectric materials.

## 3. Modulation Doping

**Figure 5:**
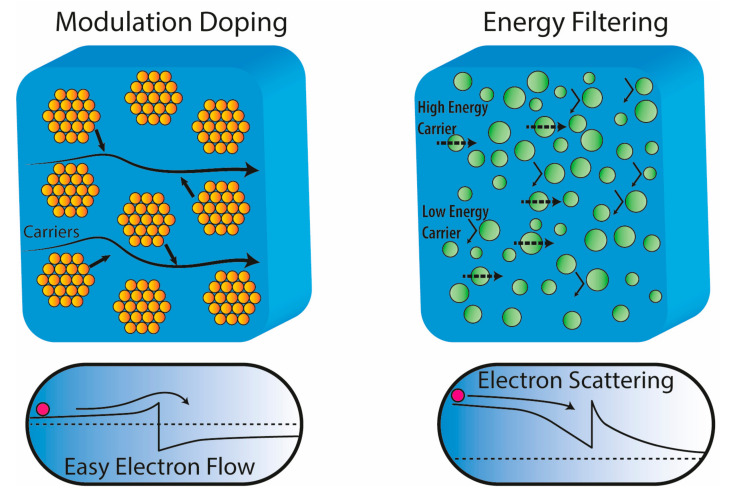
Comparison between a modulated doped semiconductor and a multiphase compound benefiting from energy filtering.

Modulation doping is a well-recognised concept to increase the conductivity in heterojunction devices [127]. The main idea behind modulation doping is to use the offset in the band structure between two semiconductors in combination with heavy doping of the material with a wider bandgap so that there is a transfer of carriers from the wide bandgap to the narrow bandgap material. The transferred carriers create two-dimensional electron gas, and they are essentially separated from the donor phase, which consequently increases the charge carrier mobility [128]. Conventionally, this strategy was employed to create *p*-channel devices, called modulation-doped field-effect transistors (MODFET) [129]. The difference between energy filtering and modulated doped samples is shown schematically in Figure 5. In the case of modulation doping, the secondary phase increases the conductivity by donating electrons to the host semiconductor, while in the case of energy filtering, the secondary phase scatters electrons and reduces mobility.

The thermoelectric research community has also used this mechanism to enhance the thermoelectric performance of materials [15,23,25,29,38]. For thermoelectric materials, a combination of two effects has enhanced the thermoelectric efficiency in the modulated doped materials: firstly, a large increase in the electrical conductivity and mobility of the charge carriers [130], and secondly, a reduction in the lattice thermal conductivity as a result of the scattering of phonons by nanostructuring [23,131].

Some attempts have also been made to explore the possibility of using modulation doping in structures similar to field-effect transistors (FETs) [129]. In this adopted structure, the thermoelectric semiconductor nanowire (channel) is enclosed by the heavily doped layer (gate) [132,133,134,135,136]. A modest increase in the power factor was achieved by this approach.

Table 2 summarises the sample compositions, fabrication methods, and corresponding references of recent studies that employed modulation doping to enhance the thermoelectric performance of multiphase materials.

## 4. Phonon Scattering

When it comes to designing a thermoelectric material, the main goal is to maintain a high electrical conductivity while, at the same time, reduce the thermal conductivity to reach an amorphous solid [147]. In semiconductors, phonon transport plays a significant role in thermal conductivity [148,149]. Increasing phonon scattering has, therefore, proven to be a key strategy to improve the efficiency of thermoelectric materials [150]. This is mainly performed by nanostructuring of the material [151], introducing grains with sizes larger than the mean free path of the charge carriers but smaller than the mean free path of the phonons [152], alloy scattering with additional mass or strain fluctuation [153,154,155], nanocomposites [156,157,158,159], and embedding interfaces by creating texture between the two materials [160].

In thermoelectric materials, the phonon-scattering mechanisms are assessed using models for the total thermal conductivity. Traditional models are from Klemens [161], Holland [162], and Callaway [163]. Impurity scattering, boundary scattering, three-phonon normal process, and Umklapp process are considered in these models, and Matthiessen’s rule is employed to determine the overall relaxation time.

The Klemens model has been successfully used to evaluate the contribution of each phase on the lattice thermal conductivity [164,165]. In this model, the thermal conductivity for each type of scattering mechanism is evaluated independently, and the overall thermal conductivity is given by [161]:

(8) $$\kappa_{tot}^{-1}=\sum_{i}\kappa_{i}^{-1}$$

For phonon-point defects, the relaxation time is given by [161]:

(9) $$\tau_{I}=\frac{\Gamma V}{4\pi \nu_{s}^{3}}\omega^{4}$$

where $\nu_{s}$ is the average sound speed on the material, V is the average atomic volume, and Γ is the mass-fluctuation, phonon-scattering parameter [166]:

(10) $$\Gamma =\frac{\bar{\Delta M^{2}}}{{\bar{M}}^{2}}$$

where $\bar{M}=\frac{\sum_{n}c_{n}\sum_{i}f_{i,n}M_{i,n}}{\sum_{n}c_{n}}$, $\bar{\Delta M}=\frac{\sum_{n}c_{n}\sum_{i}f_{i,n}(M_{i,n}-\bar{M_{n}}){\;}^{2}}{\sum_{n}c_{n}}$, $\bar{M_{n}}=\sum_{i}f_{i,n}M_{i,n}$, $c_{n}$ is the stoichiometry of the n-th component and $f_{i,n}$ is the fraction of the i-th element that is presented in the n-th component. Using this formulation, the effect of the material composition can be inferred from the lattice thermal conductivity.

Effective medium approximation, on the other hand, presents a more simplified version of the formula described above. The model incorporates an interface resistance, called Kapitza resistance ($R_{\kappa }$), in series with the inter grain resistance [167]. This model is further developed to consider the shapes, orientations, volume fractions, and thermal conductivities of the phases [160]. For instance, the thermal conductivity of a two-phase material with spherical inclusions is expressed by:

(11) $$\kappa^{*}=\kappa_{m}\frac{\kappa_{p}\left(1+2\alpha \right)+2\kappa_{m}+2f\left[\kappa_{p}\left(1-\alpha \right)-\kappa_{r}\right]}{\kappa_{p}\left(1+2\alpha \right)+2\kappa_{m}-f\left[\kappa_{p}\left(1-\alpha \right)-\kappa_{r}\right)]}$$

where $\kappa_{m}$ is the thermal conductivity of the matrix, $\kappa_{p}$ is the thermal conductivity of the secondary phase, and f is the volume fraction of the secondary phase. The non-dimensional parameter, α, is the ratio of the Kapitza length, $L_{\kappa }$, and the second phase radius, a:

(12) $$\alpha =\frac{L_{\kappa }}{a}=\frac{\kappa_{m}R_{\kappa }}{a}$$

**Figure 6:**
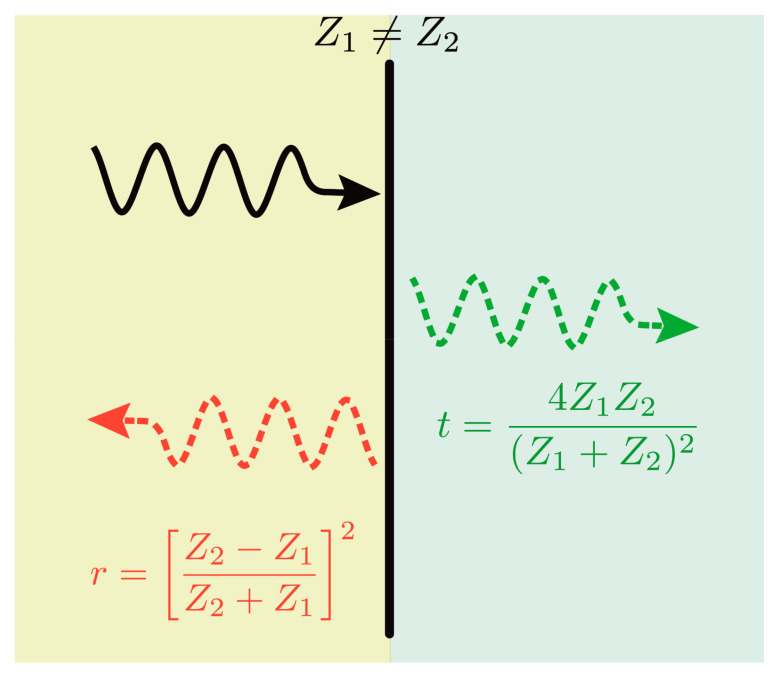
Phonon transmission and reflection due to impedance mismatch.

An even more simple model than that which has been used to describe the scattering in multiphase materials is impedance mismatch [152]. The specific acoustic impedance of a material is the analogue of the electrical impedance for electrical circuits. In this case, the acoustic impedance measures the opposition of a system when acoustic pressure is applied to it and its calculated as:

(13) Z=ρν

where ρ is the volumetric density of the medium and $\nu_{S}$ is the speed of the sound in the medium. At the interface of two materials, the reflection (r) and transmission (t) energy coefficients are [168]:

(14) $$r={\left[\frac{Z_{2}-Z_{1}}{Z_{2}+Z_{1}}\right]}^{2},\;t=\frac{4Z_{1}Z_{2}}{{\left(Z_{1}+Z_{2}\right)}^{2}}$$

where $Z_{1}$ and $Z_{2}$ are the acoustic impedance of the two materials. Figure 6 illustrates phonon transmission and reflection between two dissimilar materials.

Experimentally, impedance mismatch between phases has been shown to reduce the thermal conductivity of the bulk material. The impedance mismatch between the PbTe and PbS rich phases in (Pb_0.95_Sn_0.05_Te)_1−*x*_(PbS)*_x_* samples led to an inhibition of the heat flow, with the lattice thermal conductivity reaching 0.4 W·m^−1^·K^−1^ for the sample with 8% PbS, an 80% reduction in the reported values for the bulk material [169].

In general, phonon scattering has proven to be an effective strategy to reduce the lattice thermal conductivity in multiphase lead telluride-based materials [170,171,172,173] and bismuth telluride-based [174,175,176] materials. For instance, nano-engineered multiphase PbTe—*x*% InSb compounds showed an exceptionally low minimum lattice thermal conductivity of ~0.3 W·m^−1^·K^−1^ at ~770 K for 4% InSb and consequently a *z*T value of ~1.83 at 770 K [177]. Even higher *z*T values of ~2 were observed for Pb_(1−*x*)_Na*_x_*Te_0.65_S_0.25_Se_0.1_ compounds [30,31], where the combined effects of phonon scattering at nanoprecipitates and the increase in the power factor due to heterogeneous distribution of dopants between phases were recognised to be responsible for the high TE efficiency. Nano and micro-sized precipitates in Pb_1−*x*_Ga*_x_*Te (*x* = 0.01, 0.02, 0.03, and 0.04) compounds have shown a reduction in the lattice thermal conductivity, reaching 1.6 W·m^−1^·K^−1^. A larger fraction of a secondary phase with high thermal conductivity can increase the total thermal conductivity, reported for PbTe-Ge*_x_* [178]. Five per cent of GeTe reduced the lattice thermal conductivity to 1.1 W·m^−1^·K^−1^, while a sample with *x* = 0.2 showed a lattice thermal conductivity similar to the pristine sample.

Adding a secondary phase of TiO_2_ to a Bi_2_Se_3_ host increased the Seebeck coefficient by energy filtering and simultaneously reduced the lattice thermal conductivity by 45% [101]. The lattice thermal conductivity of multiphase half-Heusler (Hf_0.6_Zr_0.4_)NiSn_0.99_Sb_0.01_ material was reduced from ~4.5 W·m^−1^·K^−1^ for the pristine sample to ~2.9 W·m^−1^·K^−1^ for the sample with 20wt.% of tungsten at 300 K [112]; the combined effects of phonon scattering and energy filtering due to the presence of metallic tungsten improved the *z*T by 55%.

**Figure 7:**
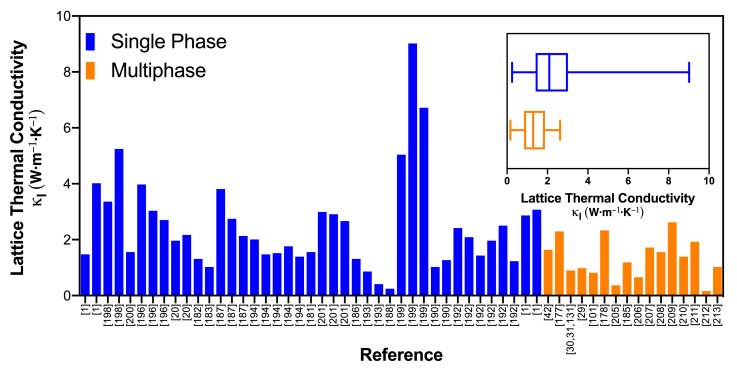
The lattice thermal conductivity of multiphase thermoelectric materials compared with single-phase counterparts; data extracted from [1,20,29,30,31,42,101,131,177,178,182,183,184,185,186,187,188,189,190,191,192,193,194,195,196,197,198,199,200,201,202,203,204,205,206,207,208,209,210,211,212,213]. The inset shows a boxplot of the same data.

The lattice thermal conductivity values of several single-phase chalcogenides are compared with their multiphase counterparts in Figure 7. The data for single-phase materials were manually extracted from the Materials Research Laboratory Energy Materials Datamining website [179,180]. This dataset contains information of 573 thermoelectric materials from various combinations of host materials and dopants along with several thermoelectric properties measured experimentally at 300 K, 400 K, and 700 K. Here, we have selected the lattice thermal conductivity of chalcogenides, measured at 300 K. Where the lattice thermal conductivity was not available, its value was evaluated using the Wiedemann–Franz law and the Sommerfeld limit for the Lorenz number [181]. Both the bar and boxplot show that the multiphase materials consistently present lower values of the lattice thermal conductivity. In particular, the boxplot shows that the multiphase materials have, on average, lower values of lattice thermal conductivity.

## 5. Models to Estimate the Transport Properties

In general, multiphase materials can increase phonon scattering, increase the Seebeck coefficient due to energy filtering, and increase electrical conductivity due to modulation doping. These micro and nano effects clearly influence the material on a macroscale. It is, however, of interest to have simplified expressions to predict the behaviour of these materials from the composition and electronic band engineering perspectives. Regarding the thermal conductivity, the effective medium theory allows us to approximate the effective Seebeck coefficient and the electrical conductivity of multiphase materials [214]. There are two main equations to evaluate these properties. The most common equation is the one derived from the usual effective mean theory [215]:

(15) $$\sum_{i}v_{i}\frac{\zeta_{i}-\zeta }{\zeta_{i}+2\zeta }=0$$

where $v_{i}$ and $\zeta_{i}$ are the volume fraction and property of phase i, respectively, and ζ is the effective material property. The electrical conductivity can be calculated by setting ζ=σ, and the Seebeck coefficient can be calculated by setting ζ=S/σ [216]. The second equation is based on the generalised effective mean theory:

(16) $$\sum_{i}v_{i}\frac{\zeta_{i}^{\frac{1}{t}}-\zeta^{\frac{1}{t}}}{\zeta_{i}^{\frac{1}{t}}+A\cdot \zeta^{\frac{1}{t}}\;}=0$$

where t is a measure of the grain structure and morphology, and $A=\left(1-p_{c}\right)/p_{c}$, where $p_{c}$ is the percolation threshold. Both parameters can be determined by fitting experimental data, and $p_{c}$ is estimated from the lattice type and dimensions of the network [217]. The Seebeck coefficient and the conductivity are estimated similar to the previous equation [218].

Recently, models based on electrical networks have been introduced to estimate the electronic properties [214,219,220] and finite element analysis [221]. These latter models divide the material into pixels (for a 2D analysis) or voxels (for a 3D analysis) grids, where each node corresponds to a fraction of the total volume. Each voxel/pixel is required to be larger than the mean free paths of the carriers and phonons so that the transport is diffusive. Each node is connected to its neighbours by a resistance that is an electric resistance (to calculate the electrical conductivity) and a thermal resistance (to calculate the thermal conductivity) in series with an interface resistance if needed. Following the construction of grids, a nodal analysis will be conducted [222] to determine the temperature and voltage profile of the grids. The Seebeck coefficient is estimated by assigning each node in the electric grid to a local voltage source in series, representing the local Seebeck voltage. By using a Norton equivalent of the voltage source [223], the usual nodal formulation can be applied, and the bulk Seebeck coefficient will be estimated. The general expression is presented as [220]:

(17) $$0=\sum_{k\neq l}V_{kl}G_{kl}+\sum_{k\neq l}I_{kl}$$

where $V_{k,l}$ is the voltage (or temperature) difference between nodes k and l, $I_{k,l}$ is the current flowing between nodes k and l, and $G_{k,l}$ is the conductivity between nodes k and l. The conductivity will be determined by the local thermoelectric properties of the voxels or pixels:

(18) $$\sigma_{kl}^{-1}=\sigma_{k}^{-1}+\sigma_{l}^{-1},\;\kappa_{kl}^{-1}=\kappa_{k}^{-1}+\kappa_{l}^{-1}$$

where $\sigma_{k},\;\sigma_{l}$ is the electrical conductivity of nodes k and l, respectively, and $\kappa_{k},\;\kappa_{l}$ is the thermal resistivity of nodes k and l.

The main advantage of this approach is that this linear system of equations can easily be solved by electric circuit solvers, and the results can be directly interpreted by engineers. However, the quantum effects are not approximated by an effective medium theory in this model, and therefore, it provides similar accuracy to the more simple models described earlier [219]. Overall, from a design perspective, these equations suggest that the incorporation of highly conductive phases (both thermally and electrically) results in an overall increase in the conductivity of materials. This effect has been experimentally proven in several materials. Graphene, a zero-bandgap material [224], has been successfully used to create thermoelectric materials with high conductivity [225,226,227], the electrical conductivity of CoSb_3_ reached 1.2 × 10^6^ S/m, four times larger than the pristine sample at 300 K [226]. Copper telluride in Bi_0.5_Sb_1.5_Te_3_ [228] and a composite of ZnSb matrix with minority phases of Zn_4_Sb_3_, Zn_3_P_2_, and Cu_5_Zn_8_ [229] have shown higher electrical conductivity. It should be noted that the values of electronic transport properties are greatly dependent on the fabrication methods [230], quality of the raw materials, and oxidisation of the material [231,232,233].

High-temperature, oxide-based thermoelectric materials have also been shown to benefit from the presence of secondary phases. A multiphase compound of Ca_3_Co_4_O_9_, matrimid polymer + Ag, and carbon black [40] showed a reduction in the electrical conductivity compared to the porous Ca_3_Co_4_O_9_, while the highly conductive phase of Ag reduced a significant deterioration of conductivity.

## 6. Magnetic Effects

The effects of magnetism on the performance of thermoelectric materials have been investigated for some time, but with less detailed analysis. The idea of spin-wave scattering and magnon drag was proposed in the middle of the last century, where magnon scattering was shown to contribute to an increase in the Seebeck coefficient [234]. Magnons are bosonic quasiparticles, the quanta associated with spin waves [235]. When a magnetic material is subjected to a temperature gradient, the hotter side contains a higher density of magnons that will diffuse towards the cooler side; this magnon flux “drags” the free charge carriers due to the electron–magnon collisions and gives rise to a second contribution to the Seebeck coefficient, called the magnon thermopower [236]. A hydrodynamical, Galilean based expression for the magnon thermopower is [237]

(19) $$S_{md}=\frac{2}{3}\cdot \frac{C_{m}}{n_{e}e}\cdot \frac{1}{1+\frac{\tau_{em}}{\tau_{m}}}$$

where $C_{m}$ is the magnon specific heat capacity per unit volume, $\tau_{m}$ and $\tau_{em}$ are, respectively, the transport mean-free time for the electron and magnon–electron collision, e is carrier charge, and $n_{e}$ is the charge carrier density.

When dealing with magnetism in semiconductors, three main strategies have been proposed to improve the thermoelectric efficiency: (1) optimise thermoelectric properties of magnetic materials using strategies known for non-magnetic materials [43,44,45,238,239]; (2) introduce a magnetic dopant in a non-magnetic material [46,47,48,240,241,242,243]; (3) introduce a magnetic secondary phase in a non-magnetic material [49,50].

### 6.1. Magnetic Semiconductors

Examples of magnetic semiconductors are FeSb_2_ [244,245], MnTe [246,247,248], Cr_2_Ge_2_Te_6_ [249], MgAgSb [250], MnSe [251], and FeSe [252]. Experimental results have shown their potential as thermoelectric materials; for instance, a massive Seebeck coefficient of ~27 mV/K has been reported for FeSb_2_ (albeit at a low temperature of ~12 K) [253]. It is not easy, however, to establish a causal relationship between magnetism and the Seebeck coefficient. A common method is to check whether a heavily doped sample with a high carrier concentration shows a large value of the Seebeck coefficient [254], because this indicates a possible effect of electron–magnon scattering, which increases the Seebeck coefficient. Fitting mathematical models to the experimental data has also been proposed as a method to identify the magnetic thermopower [255]. Clearly, measuring the transport properties as a function of the magnetic field is the best method to determine the magnetic thermopower [236,256,257].

A spin-dependent Seebeck coefficient can occur in magnetic semiconductors [258]; since the Seebeck coefficients for the two spin channels of spin-up (S^↑^) and spin-down (S^↓^) are not equal, a spin current proportional to the difference between S^↑^ and S^↓^ flows through the magnetic material even in the absence of a charge current [259]. The literature refers to spintronics as the field of study that investigates devices that exploit the properties of electrons spins. In thermoelectricity, this is known as spin caloritronic [260]. This new field of research has attracted the interest of the thermoelectric research community [261,262,263].

### 6.2. Magnetic Dopants in Non-Magnetic Semiconductors

Doping non-magnetic thermoelectric materials with magnetic elements has improved the power factor. Magnetic doping of CuGaTe_2_ with manganese ions (Mn^2+^) increased the effective mass of the carriers due to the interaction of the magnetic ions and the charge carriers and, consequently, increased the Seebeck coefficient [264]. This effect has also been reported for a Chromium doped Bi_2_Te_3_ [265]. The negative side effect of magnetic ion dopants is the decrease in the charge carrier mobility that results in a reduction in the electrical conductivity. Overall, the power factor is shown to be increased [266,267,268].

### 6.3. Secondary Magnetic Phases

The natural extension of using magnetic elements is to include magnetic phases to enhance the performance of thermoelectric materials [264,269,270]. The magnetic particles of BaFe_12_O_19_ in Ba_0.3_In_0.3_Co_4_Sb_12_ formed a magnetic composite; BaFe_12_O_19_ nanoparticles trap electrons in the ferromagnetic phase due to the spiral motion of the electrons generated by non-uniform spherical magnetic fields. This effectively suppresses the deterioration of thermoelectric efficiency in the intrinsic excitation region [271]. In the paramagnetic phase (at temperatures above the Curie temperature), though, the nanoparticles release the trapped electrons to increase the carrier concentration in the intrinsic excitation. This effect enhances the overall performance of the thermoelectric material.

Coherent magnetic full-Heusler nanoparticles (Ti(Ni_4/3_Fe_2/3_)Sn) in a half-Heusler matrix (Ti_0.25_Zr_0.25_Hf_0.5_NiSn_0.975_Sb_0.025_) showed significant enhancements of both carrier mobility and the Seebeck coefficient [272]. The magnetic nanoparticles interact with the spin of itinerant carriers, leading to charge localisation (which consequently leads to a decrease in the charge density) and the formation of overlapping bound magnetic polarons (that leads to an increase in mobility).

Interestingly, some magnetic phenomena only occur when the particles are smaller than a certain size [273]. Nanoparticles provide a good platform to take advantage of these magnetic phenomena for the purpose of optimising the thermoelectric performance of materials. If the size of a ferromagnetic nanoparticle is small enough to have only several magnetic domains, it can be magnetised similar to a paramagnet under an external magnetic field, except with a much greater magnetisation. This mechanism is known as superparamagnetism (magnetisation of the nanoparticles can randomly flip direction under the influence of temperature, and they can be magnetised similar to a paramagnet under an external magnetic field [274]). Experimentally, soft magnet transition metals (Fe, Co, or Ni) nanoparticles were embedded in a Ba_0.3_In_0.3_Co_4_Sb_12_ matrix [275]. The superparamagnetism fluctuations of the nanoparticles gave rise to multiple scattering of electrons and enhanced phonon scattering. These effects increased the overall thermoelectric efficiency of the material.

In general, secondary magnetic phases introduce a new degree of freedom to enhance thermoelectric materials. The effects of magnetism in semiconductors are not fully understood yet, and general expressions for the contribution of the magnon-drag on the Seebeck coefficient are unavailable yet. The need for more experimental and theoretical investigation presents an opportunity for thermoelectricity.

Table 3 summarises the sample composition and fabrication techniques of recent studies that reported magnetic effects in thermoelectric materials.

**Figure 8:**
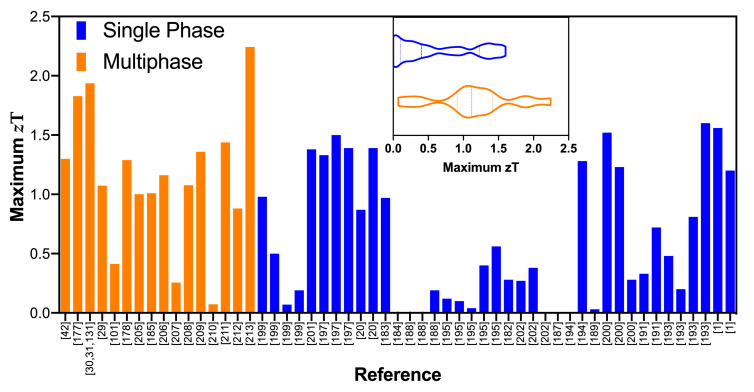
*z*T values of multiphase thermoelectric materials compared with single-phase counterparts; data extracted from [1,20,29,30,31,42,101,131,177,178,182,183,184,185,186,187,188,189,190,191,192,193,194,195,196,197,198,199,200,201,202,203,204,205,206,207,208,209,210,211,212,213]. The inset shows the truncated violin plot of the same data, where the dashed line represents the median and dotted lines represent the quartiles.

Figure 8 compares the maximum *z*T values reported for several single chalcogenides with their multiphase counterparts. The data for single-phase materials were manually extracted from the Materials Research Laboratory Energy Materials Datamining website [179,180]. Both the bar and violin plot show that the multiphase materials consistently present larger values of *z*T. In particular, the violin plot shows that many single-phase materials have low *z*T, while the *z*T for multiphase materials is distributed towards larger values.

## 7. Summary and Outlook

Here, we have reviewed the principal mechanisms and strategies employed to enhance the thermoelectric efficiency of multiphase thermoelectric materials. The interfaces between phases in a material can be utilised to construct potential barriers in the electronic band structure and perform an energy filtering on the charge carriers, where the low energy charge carriers will be filtered/scattered. This can increase the average energy at which the electronic conduction occurs and consequently increase the Seebeck coefficient. The effect of the barrier height can be estimated from the relaxation time approximation from the Boltzmann equation. For a metallic secondary phase, the potential barrier height can be estimated from the work function of the metal and electron affinity of the semiconductor following Schottky’s rule. For heterojunctions (two different semiconductors), the potential barrier is proportional to the difference of the electron affinities between the two materials and can be estimated from Anderson’s rule. Experimental results have shown that the energy-filtering effect can increase the Seebeck coefficient while decreasing electrical conductivity. The overall result, however, is an increase in the power factor.

Modulation doping is a well-known technique for enhancing the conductivity of semiconductor devices. In this paper, we briefly reviewed the mechanism by which modulation doping is used in multiphase thermoelectric materials.

An increase in phonon scattering due to the presence of secondary phases improves the thermoelectric efficiency of these materials. Secondary phases decrease the relaxation time of the phonons. Traditional models can be employed to verify the effect of a secondary phase on the thermal conductivity of materials. The Klemens model has been used to theoretically explain the reduction in thermal conductivity. The effective medium approximation has been promising in describing the phonon scattering mechanisms of materials with simple structures, such as spherical particles. In an even more simplistic view, for some materials, the transmission and reflection coefficients determined based on the acoustic impedance of the primary and secondary phases can be readily used to evaluate the effect of multiple phases on the reduction of thermal conductivity. Experimentally, a large increase in the *z*T has been reported with the use of secondary phases.

Mathematical models based on effective medium theory and networks can also estimate the Seebeck coefficient and electrical conductivity of multiphase materials. These models can be used as a tool to evaluate and tune the properties of thermoelectric materials.

The incorporation of magnetic doping elements and secondary phases in thermoelectric materials has introduced new possibilities to enhance their efficiency. The scattering of electrons due to interactions between charge carriers and magnons can contribute to an increase in the Seebeck coefficient. A magnon flux generated from a temperature gradient “drags” the free carriers and contributes positively to the Seebeck coefficient—this effect is called the magnon thermopower. Experimentally, this has proven to be a promising strategy to enhance the thermoelectric properties of several materials.

Overall, multiphase materials have been shown to be instrumental in achieving high thermoelectric efficiency. Multiple phases allow for more degrees of freedom in the materials design as each phase can be finely tuned to improve selected properties of the multiphase material.

## Figures and Tables

**Table 1:** Compositions, fabrication methods, and corresponding references of multiphase materials that benefited from the energy-filtering effect.

Composition	Fabrication Technique	Ref.
Bi_0.5_Sb_1.5_Te + (0, 1.0, 2.0, 4.0, and 6.0 wt.%) nanoparticles of Sb_2_O_3_	Casting (Bi_0.5_Sb_1.5_Te) + ball milling of Bi_0.5_Sb_1.5_Te and commercial powder of Sb_2_O_3_+ sintering	[113]
Bi_2_Te_2.7_Se_0.3_ powder + ~2 nm layer of film of ZnO	Solution-based synthesis of Bi-Te-Se powder + atomic layer deposition of ZnO + hot pressing	[114]
Bi_1−x_Sb_x_ (*x* = 0, 0.10, 0.12, 0.13, 0.14, 0.2) + 3 wt.% carbon nanotubes	Ball milling + cold pressing + annealing	[115]
Bi_0.46_Sb_1.54_Te_3_ + (0, 0.1, 0.2, and 0.3 wt.%) SiC	Ball milling + sintering	[70]
Bi_0.4_Sb_1.6_Te_3_ + (0.1%, 0.2%, and 0.3 wt.%) CuInTe_2_	Casting each phase separately + ball milling + sintering	[116]
Lu_0.1_Bi_1.9_Te_3_ + (0, 0.0, 0.05, 1) wt.% carbon nanotubes	Hydrothermal synthesis + grinding + hot pressing	[117]
Bi_0.5_Sb_1.5_Te_3_ + (0, 0.1, 0.2, 0.3, 0.4 wt.%) BaTiO_3_	Hydrothermally synthesised BaTiO_3_ + Commercial ingots of Bi_0.5_Sb_1.5_Te_3_ were grinded and sintered	[74]
Bi_0.5_Sb_1.5_Te_3_ + SrTiO_3_	Bi_0.5_Sb_1.5_Te_3_ films were grown on SrTiO_3_ substrates by co-sputtering	[118]
Bi_2_Te_3_ + Bi_0.5_Sb_1.5_Te_3_ thin films	Radio-frequency magnetron sputtering of Bi_2_Te_3_/Bi_0.5_Sb_1.5_Te_3_ layers on a SiO_2_/Si(001) substrate	[119]
Bi_0.4_Sb_1.6_Te_3_ + (0, 0.2, 0.4, and 0.6 vol.%) CuGaTe_2_	Vacuum melting + hot pressing	[120]
Bi_0.5_Sb_1.5_Te_3_ + 2 wt.% (Gd_2_O_3_, Gd_1.98_Bi_0.02_O_3_)	Powders for each phase were prepared by induction melting then mixed by spray pyrolysis + sintering	[121]
Bi_2_Te_3_ + (1, 2, and 5 wt.%) SnS	Commercial powders were mixed, cold pressed, and annealed	[122]
Bi_0.3_Sb_1.7_Te_3_ + (0, 0.25, 0.50, and 0.75 wt.%) TiC	Ball milling + sintering	[123]
Bi_2_Te_3_ + ~4 wt.% of Cu_1.5_Te	Solution-based synthesis (each phase separately) + hot pressing	[124]
Coated grains of SnTe with CuInTe_2_	CuInTe_2_ was formed by cation exchange of Sn by Cu and In on the surface of ball-milled SnTe powder	[125]
Bi_0.5_Sb_1.5_Te_3_-Cu_0.07_ + (0, 0.5, and 1.0 wt.%) HfO_2_	Water atomisation + ball milling + sintering	[126]
SiGe + (2, 4, 6, 8, 10 wt.%) TiB_2_	Ball milling + hot pressing	[108]

**Table 2:** Sample compositions and fabrication methods of references that employed modulation doping to enhance the thermoelectric performance of multiphase materials.

Composition	Fabrication Method	Ref.
Ba_8_(Al*_x_*Ga_1−*x*_)_16_Ge_30_ (*x* = 0, 0.20, 0.23, 0.25, 0.33, 0.50, and 1)	Casting (each phase separately) + ball milling + sintering	[137]
AgBiSe_2_ + Bi_4_Se_3_		[138]
Cu_2_SnS_3_ + (0, 1, 3, and 5% mol) CuCo_2_S_4_	Casting (each phase separately) + ball milling + sintering	[25,139]
Si*_a_*(Mg_2_Si + *x* at. % Bi)_1−*a*_ (*a* = 0.39, 0.50, 0.56, 0.59, and 0.67; *x* = 0.3, 0.8, 1.3, 1.8, 2.5)	Bi-doped Mg_2_Si fabricated using induction melting + melt spinning si + sintering	[140]
*p*-type organic conducting polymer PEDOT:PSS + Ge	PEDOT:PSS coated with Ge layer	[141]
(Ge_2_Te_2_)_x_(CuInTe_2_)_1−*x*_ (*x* = 98, 95, 90, 87.5, 85, 70, 30, and 10%)	Casting + hand milling + hot pressing	[142]
BiCuSeO + Bi_0.8_Pb_0.2_Cu_0.8_Ni_0.2_SeO	Each phase was fabricated by Mechanical alloying + ball milling of mixture + sintering	[143]
BiCuSeO + Bi_0.8_Er_0.2_CuSeO	Each phase was fabricated by ball milling + sintering. The final composition was obtained by ball milling + sintering	[144]
BiCuSeO + Bi_0.8_Ba_0.2_CuSe_0.8_Te_0.2_O	Each phase was fabricated by mechanical alloying + milling the mixture + sintering	[145]
Pb_(1−*x*)_Na*_x_*Te_0.65_S_0.25_Se_0.1_ (*x* = 0.005, 0.01, 0.0015, 0.02, 0.025, and 0.03)	Casting PbSe and PbS + mixing stoichiometric amounts of PbSe, PbS, Pb, Te, and Na (casting) + sintering	[29]
Pb_0.97_Na_0.03_Te_(1−*x)*_S*_x_* (*x* = 0.1, 0.15, 0.2, 0.25, 0.3, and 0.35)	Casting + hand milling + sintering	[146]

**Table 3:** Sample composition, fabrication technique, and corresponding references which studied magnetic effects in thermoelectric materials.

System	Fabrication Method	Type	Ref.
CuGa_1−*x*_Mn*_x_*Te_2_ (*x* = 0, 0.01, 0.02, and 0.03)	Casting + hand milling + sintering	Magnetic dopant	[264]
Bi_2−*x*_Cr*_x_*Te_3_ (*x* = 0, 0.01, 0.02, 0.05, and 0.10)	Casting + hand milling + sintering	Magnetic dopant	[265]
Fe_3−*x*_Ti*_x_*Sn (*x* = 0, 0.25, 0.5, 0.75)	Casting	Magnetic material	[239]
Sn_1.03−*x*_Mn*_x_*Te (*x* = 0, 0.05, 0.07, and 0.1)	Casting + cold pelletising	Magnetic dopant	[266]
Ba_0.3_In_0.3_Co_4_Sb_12_ +*x* BaFe_12_O_19_) (*x* = 0.15%, 0.25%, 0.35%, and 0.45%)	Ball milling + sintering	Magnetic phase	[271]
Ti_0.25_Zr_0.25_Hf_0.5_(Ni,Fe*_x_*)Sn_0.975_Sb_0.025_ (*x* = 0, 0.05, 0.01, 0.15)	Casting + hand milling + sintering	Magnetic phase	[272]
Mn_1−*x*_Na*_x_*Se (0 ≤ *x* ≤ 0.03)	Ball milling + annealing + hot pressing	Magnetic material	[251]
FeSb_2_	Hand milling + annealing + hot pressing	Magnetic material	[244]

## References

[1] Biswas K., He J., Blum I.D., Wu C.I., Hogan T.P., Seidman D.N., Dravid V.P., Kanatzidis M.G. (2012). High-Performance Bulk Thermoelectrics with All-Scale Hierarchical Architectures. Nature.

[2] Vining C.B. (2009). An Inconvenient Truth about Thermoelectrics. Nat. Mater..

[3] Tritt T.M., Boettner H., Chen L. (2008). Thermoelectrics: Direct Solar Thermal Energy Conversion. MRS Bull..

[4] Cutler M., Mott N.F. (1969). Observation of Anderson Localization in an Electron Gas. Phys. Rev..

[5] Siemens M.E., Li Q., Yang R., Nelson K.A., Anderson E.H., Murnane M.M., Kapteyn H.C. (2010). Quasi-Ballistic Thermal Transport from Nanoscale Interfaces Observed Using Ultrafast Coherent Soft X-Ray Beams. Nat. Mater..

[6] Delaire O., Ma J., Marty K., May A.F., McGuire M.A., Du M.H., Singh D.J., Podlesnyak A., Ehlers G., Lumsden M.D. (2011). Giant Anharmonic Phonon Scattering in PbTe. Nat. Mater..

[7] Hu Y., Zeng L., Minnich A.J., Dresselhaus M.S., Chen G. (2015). Spectral Mapping of Thermal Conductivity through Nanoscale Ballistic Transport. Nat. Nanotechnol..

[8] Hu L., Zhang Y., Wu H., Li J., Li Y., McKenna M., He J., Liu F., Pennycook S.J., Zeng X. (2018). Entropy Engineering of SnTe: Multi-Principal-Element Alloying Leading to Ultralow Lattice Thermal Conductivity and State-of-the-Art Thermoelectric Performance. Adv. Energy Mater..

[9] Fan Z., Wang H., Wu Y., Liu X.J., Lu Z.P. (2016). Thermoelectric High-Entropy Alloys with Low Lattice Thermal Conductivity. RSC Adv..

[10] Wei P.C., Liao C.N., Wu H.J., Yang D., He J., Biesold-McGee G.V., Liang S., Yen W.T., Tang X., Yeh J.W. (2020). Thermodynamic Routes to Ultralow Thermal Conductivity and High Thermoelectric Performance. Adv. Mater..

[11] Joshi G., Lee H., Lan Y., Wang X., Zhu G., Wang D., Gould R.W., Cuff D.C., Tang M.Y., Dresselhaus M.S. (2008). Enhanced Thermoelectric Figure-of-Merit in Nanostructured p-Type Silicon Germanium Bulk Alloys. Nano Lett..

[12] Wang X.W., Lee H., Lan Y.C., Zhu G.H., Joshi G., Wang D.Z., Yang J., Muto A.J., Tang M.Y., Klatsky J. (2008). Enhanced Thermoelectric Figure of Merit in Nanostructured N-Type Silicon Germanium Bulk Alloy. Appl. Phys. Lett..

[13] Lan Y.C., Minnich A.J., Chen G., Ren Z.F. (2010). Enhancement of Thermoelectric Figure-of-Merit by a Bulk Nanostructuring Approach. Adv. Funct. Mater..

[14] Ren Z., Poudel B.E.D., Chen G., Lan Y., Wang D., Hao Q., Dresselhaus M., Ma Y.I., Yan X., Chen X. (2014). Methods for High Figure-of-Merit in Nanostructured Thermoelectric Materials. U.S. Patent.

[15] Yu B., Zebarjadi M., Wang H., Lukas K., Wang H., Wang D., Opeil C., Dresselhaus M., Chen G., Ren Z. (2012). Enhancement of Thermoelectric Properties by Modulation-Doping in Silicon Germanium Alloy Nanocomposites. Nano Lett..

[16] Wang H., Cao X., Takagiwa Y., Snyder G.J. (2015). Higher Mobility in Bulk Semiconductors by Separating the Dopants from the Charge-Conducting Band—A Case Study of Thermoelectric PbSe. Mater. Horiz..

[17] Thesberg M., Pourfath M., Kosina H., Neophytou N. (2015). The Influence of Non-Idealities on the Thermoelectric Power Factor of Nanostructured Superlattices. J. Appl. Phys..

[18] Rhyee J.S., Lee K.H., Lee S.M., Cho E., Kim S.I., Lee E., Kwon Y.S., Shim J.H., Kotliar G. (2009). Peierls Distortion as a Route to High Thermoelectric Performance in In(4)Se(3-Delta) Crystals. Nature.

[19] Zhou J., Yang R., Chen G., Dresselhaus M.S. (2011). Optimal Bandwidth for High Efficiency Thermoelectrics. Phys. Rev. Lett..

[20] Heremans J.P., Jovovic V., Toberer E.S., Saramat A., Kurosaki K., Charoenphakdee A., Yamanaka S., Snyder G.J. (2008). Enhancement of Thermoelectric Efficiency in PbTe by Distortion of the Electronic Density of States. Science.

[21] Mao J., Liu Z., Ren Z. (2016). Size Effect in Thermoelectric Materials. npj Quant. Mater..

[22] Zeng G., Bowers J.E., Zide J.M.O., Gossard A.C., Kim W., Singer S., Majumdar A., Singh R., Bian Z., Zhang Y. (2006). ErAs:InGaAs/InGaAlAs Superlattice Thin-Film Power Generator Array. Appl. Phys. Lett..

[23] Zebarjadi M., Joshi G., Zhu G., Yu B., Minnich A., Lan Y., Wang X., Dresselhaus M., Ren Z., Chen G. (2011). Power Factor Enhancement by Modulation Doping in Bulk Nanocomposites. Nano Lett..

[24] Hou Q.R., Gu B.F., Chen Y.B., He Y.J., Sun J.L. (2014). Enhancement of the Thermoelectric Power Factor of MnSi1.7 Film by Modulation Doping of Al and Cu. Appl. Phys. A Mater..

[25] Gu Y., Ai W., Zhao Y., Pan L., Lu C., Zong P., Hu X., Xu Z., Wang Y. (2021). Remarkable Thermoelectric Property Enhancement in Cu_2_SnS_3_–CuCo_2_S_4_ Nanocomposites via 3D Modulation Doping. J. Mater. Chem. A.

[26] Wu L.H., Li X., Wang S.Y., Zhang T.S., Yang J., Zhang W.Q., Chen L.D., Yang J.H. (2017). Resonant Level-Induced High Thermoelectric Response in Indium-Doped GeTe. NPG Asia Mater..

[27] Zhang Q.Y., Wang H., Liu W.S., Wang H.Z., Yu B., Zhang Q., Tian Z.T., Ni G., Lee S., Esfarjani K. (2012). Enhancement of Thermoelectric Figure-of-Merit by Resonant States of Aluminium Doping in Lead Selenide. Energy Environ. Sci..

[28] Kim R., Lundstrom M.S. (2012). Computational Study of Energy Filtering Effects in One-Dimensional Composite Nano-Structures. J. Appl. Phys..

[29] Yamini A., Li T., Mitchell D.R.G., Cairney J.M. (2016). Elemental Distributions within Multiphase Quaternary Pb Chalcogenide Thermoelectric Materials Determined through Three-Dimensional Atom Probe Tomography. Nano Energy.

[30] Aminorroaya Yamini S., Mitchell D.R., Avdeev M. (2016). In Situ Characterisation of Nanostructured Multiphase Thermoelectric Materials at Elevated Temperatures. Phys. Chem. Chem. Phys..

[31] Byrnes J., Mitchell D.R.G., Yamini S.A. (2020). Thermoelectric Performance of Thermally Aged Nanostructured Bulk Materials-a Case Study of Lead Chalcogenides. Mater. Today Phys..

[32] Gao Y.-H., Chen H., Liu N., Zhang R.-Z. (2018). Criteria for Power Factor Improvement in Thermoelectric Composite. Results Phys..

[33] Hu Q., Qiu W., Chen L., Chen J., Yang L., Tang J. (2021). Realize High Thermoelectric Properties in N-Type Bi_2_Te_2.7_Se_0.3_/Y_2_O_3_ Nanocomposites by Constructing Heterointerfaces. ACS Appl. Mater. Interfaces.

[34] Pham A.T.T., Vo P.T.N., Ta H.K.T., Lai H.T., Tran V.C., Doan T.L.H., Duong A.T., Lee C.T., Nair P.K., Zulueta Y.A. (2021). Improved Thermoelectric Power Factor Achieved by Energy Filtering in ZnO: Mg/ZnO Hetero-Structures. Thin Solid Films.

[35] Park W., Hwang H., Kim S., Park S., Jang K.-S. (2021). Optimized Thermoelectric Performance of Carbon Nanoparticle–Carbon Nanotube Heterostructures by Tuning Interface Barrier Energy. ACS Appl. Mater. Interfaces.

[36] Lee M.H., Yun J.H., Kim G., Lee J.E., Park S.-D., Reith H., Schierning G., Nielsch K., Ko W., Li A.-P. (2019). Synergetic Enhancement of Thermoelectric Performance by Selective Charge Anderson Localization–Delocalization Transition in n-Type Bi-Doped PbTe/Ag_2_Te Nanocomposite. ACS Nano.

[37] Wang C., Lin S., Chen H., Zhao Y., Zhao L., Wang H., Huo D., Chen X. (2015). Thermoelectric Performance of Si_80_Ge_20−x_Sb_x_ Based Multiphase Alloys with Inhomogeneous Dopant Distribution. Energy Convers. Manag..

[38] Wu D., Pei Y., Wang Z., Wu H., Huang L., Zhao L.-D., He J. (2014). Significantly Enhanced Thermoelectric Performance in N-Type Heterogeneous BiAgSeS Composites. Adv. Funct. Mater..

[39] Ahmad K., Wan C., Al-Eshaikh M.A., Kadachi A.N. (2019). Enhanced Thermoelectric Performance of Bi_2_Te_3_ Based Graphene Nanocomposites. Appl. Surf. Sci..

[40] Wolf M., Menekse K., Mundstock A., Hinterding R., Nietschke F., Oeckler O., Feldhoff A. (2019). Low Thermal Conductivity in Thermoelectric Oxide-Based Multiphase Composites. J. Electron. Mater..

[41] Manimozhi T., Kavirajan S., Harish S., Archana J., Kamala Bharathi K., Senthil Kumar E., Navaneethan M. (2021). Anharmonicity and Low-Thermal Conductivity in the Multi-Phase Composition of Cu_3_Bi_0.75_Sb_0.25_S_3_. Mater. Lett..

[42] Wang Z., Wang G., Wang R., Zhou X., Chen Z., Yin C., Tang M., Hu Q., Tang J., Ang R. (2018). Ga-Doping-Induced Carrier Tuning and Multiphase Engineering in n-Type PbTe with Enhanced Thermoelectric Performance. ACS Appl. Mater. Interfaces.

[43] Sun P., Oeschler N., Johnsen S., Iversen B.B., Steglich F. (2009). Thermoelectric Properties of the Narrow-Gap Semiconductors FeSb_2_ and RuSb_2_: A Comparative Study. J. Phys. Conf. Ser..

[44] Sun Y., Canulescu S., Sun P.J., Steglich F., Pryds N., Schou J., Iversen B.B. (2011). Growth and Thermoelectric Properties of FeSb_2_ Films Produced by Pulsed Laser Deposition. Appl. Phys. A Mater..

[45] Tsujii N., Mori T., Isoda Y. (2014). Phase Stability and Thermoelectric Properties of CuFeS_2_-Based Magnetic Semiconductor. J. Electron. Mater..

[46] Motohashi T., Naujalis E., Ueda R., Isawa K., Karppinen M., Yamauchi H. (2001). Simultaneously Enhanced Thermoelectric Power and Reduced Resistivity of NaxCo_2_O_4_ by Controlling Na Nonstoichiometry. Appl. Phys. Lett..

[47] Wen Q., Chang C., Pan L., Li X.T., Yang T., Guo H.H., Wang Z.H., Zhang J., Xu F., Zhang Z.D. (2017). Enhanced Thermoelectric Performance of BiCuSeO by Increasing Seebeck Coefficient through Magnetic Ion Incorporation. J. Mater. Chem. A.

[48] Xiao C., Li K., Zhang J.J., Tong W., Liu Y.W., Li Z., Huang P.C., Pan B.C., Su H.B., Xie Y. (2014). Magnetic Ions in Wide Band Gap Semiconductor Nanocrystals for Optimized Thermoelectric Properties. Mater. Horiz..

[49] Liu K.G., Li J. (2011). Thermoelectric Properties of Bulk FeSb_2_ and the Composite of FeSb_2_ and CoSb_3_ Prepared by Sintering. Appl. Mech. Mater..

[50] Tan G., Shi F., Hao S., Chi H., Bailey T.P., Zhao L.D., Uher C., Wolverton C., Dravid V.P., Kanatzidis M.G. (2015). Valence Band Modification and High Thermoelectric Performance in SnTe Heavily Alloyed with MnTe. J. Am. Chem. Soc..

[51] Hébert S., Daou R., Maignan A., Das S., Banerjee A., Klein Y., Bourgès C., Tsujii N., Mori T. (2021). Thermoelectric Materials Taking Advantage of Spin Entropy: Lessons from Chalcogenides and Oxides. Sci. Technol. Adv. Mater..

[52] Xing L., Cui W., Sang X., Hu F., Wei P., Zhu W., Nie X., Zhang Q., Zhao W. (2021). Enhanced Thermoelectric Performance and Atomic-Resolution Interfacial Structures in BiSbTe Thermo-Electro-Magnetic Nanocomposites Incorporating Magnetocaloric LaFeSi Nanoparticles. J. Mater..

[53] Ioffe A.F., Stil’bans L.S., Iordanishvili E.K., Stavitskaya T.S., Gelbtuch A., Vineyard G. (1959). Semiconductor Thermoelements and Thermoelectric Cooling. Phys. Today.

[54] Popescu A., Woods L.M., Martin J., Nolas G.S. (2009). Model of Transport Properties of Thermoelectric Nanocomposite Materials. Phys. Rev. B.

[55] Medlin D.L., Snyder G.J. (2009). Interfaces in Bulk Thermoelectric Materials: A Review for Current Opinion in Colloid and Interface Science. Curr. Opin. Colloid Interface Sci..

[56] Heremans J.P., Thrush C.M., Morelli D.T. (2004). Thermopower Enhancement in Lead Telluride Nanostructures. Phys. Rev. B.

[57] Kishimoto K., Yamamoto K., Koyanagi T. (2003). Influences of Potential Barrier Scattering on the Thermoelectric, Properties of Sintered n-Type PbTe with a Small Grain Size. Jpn. J. Appl. Phys..

[58] Hu Q.J., Zhang Y., Zhang Y.W., Li X.J., Song H.Z. (2020). High Thermoelectric Performance in Cu_2_Se/CDs Hybrid Materials. J. Alloy. Compd..

[59] Shakouri A., Lee E.Y., Smith D.L., Narayanamurti V., Bowers J. (1998). Thermoelectric Effects in Submicron Heterostructure Barriers. Microscale Thermophys. Eng..

[60] Bahk J.H., Bian Z.X., Shakouri A. (2013). Electron Energy Filtering by a Nonplanar Potential to Enhance the Thermoelectric Power Factor in Bulk Materials. Phys. Rev. B.

[61] Rowe D.M., Min G. (1994). Multiple Potential Barriers as a Possible Mechanism to Increase the Seebeck Coefficient and Electrical Power Factor. AIP Conf. Proc..

[62] Whitlow L.W., Hirano T. (1995). Superlattice Applications to Thermoelectricity. J. Appl. Phys..

[63] Zianni X., Narducci D. (2015). Parametric Modeling of Energy Filtering by Energy Barriers in Thermoelectric Nanocomposites. J. Appl. Phys..

[64] Ziman J.M. (2001). Electrons and Phonons: The Theory of Transport Phenomena in Solids.

[65] Jeong C., Kim R., Luisier M., Datta S., Lundstrom M. (2010). On Landauer versus Boltzmann and Full Band versus Effective Mass Evaluation of Thermoelectric Transport Coefficients. J. Appl. Phys..

[66] Faleev S.V., Leonard F. (2008). Theory of Enhancement of Thermoelectric Properties of Materials with Nanoinclusions. Phys. Rev. B.

[67] Gayner C., Amouyal Y. (2019). Energy Filtering of Charge Carriers: Current Trends, Challenges, and Prospects for Thermoelectric Materials. Adv. Funct. Mater..

[68] Singha A., Muralidharan B. (2017). Incoherent Scattering Can Favorably Influence Energy Filtering in Nanostructured Thermoelectrics. Sci. Rep..

[69] Narducci D., Selezneva E., Cerofolini G., Frabboni S., Ottaviani G. (2012). Impact of Energy Filtering and Carrier Localization on the Thermoelectric Properties of Granular Semiconductors. J. Solid State Chem..

[70] Zhang D., Lei J., Guan W., Ma Z., Wang C., Zhang L., Cheng Z., Wang Y. (2019). Enhanced Thermoelectric Performance of BiSbTe Alloy: Energy Filtering Effect of Nanoprecipitates and the Effect of SiC Nanoparticles. J. Alloys Compd..

[71] Liu S.Q., Kong J.H., Chen H.M., He C.B. (2019). Interfacial Energy Barrier Tuning for Enhanced Thermoelectric Performance of PEDOT Nanowire/SWNT/PEDOT:PSS Ternary Composites. ACS Appl. Energy Mater..

[72] Jiang X.Y., Zhang Q.K., Deng S.P., Zhou B., Wang B., Chen Z.Q., Qi N., Tang X.F. (2020). Enhanced Thermoelectric Performance of Polythiophene/Carbon Nanotube-Based Composites. J. Electron. Mater..

[73] An H., Pusko M., Chun D., Park S., Moon J. (2019). In-Situ Synthesis of Flexible Hybrid Composite Films for Improved Thermoelectric Performance. Chem. Eng. J..

[74] Zhang Z., Zhao W., Zhu W., Ma S., Li C., Mu X., Wei P., Nie X., Zhang Q., Zhao W. (2020). Preparation and Thermoelectric Performance of BaTiO_3_/Bi_0.5_Sb_1.5_Te_3_ Composite Materials. J. Electron. Mater..

[75] Jiang Q., Li S., Luo Y., Xin J., Li S., Li W., Zhao G., Yang J. (2020). Ecofriendly Highly Robust Ag_8_SiSe_6_-Based Thermoelectric Composites with Excellent Performance Near Room Temperature. ACS Appl. Mater. Interfaces.

[76] Cho H., Back S.Y., Yun J.H., Byeon S., Jin H., Rhyee J.S. (2020). Thermoelectric Properties and Low-Energy Carrier Filtering by Mo Microparticle Dispersion in an n-Type (CuI)_0.003_Bi_2_(Te,Se)_3_ Bulk Matrix. ACS Appl. Mater. Interfaces.

[77] Li X.X., Yu Z.K., Zhou H.B., Yang F., Zhong F., Mao X.H., Li B.Z., Xin H., Gao C.M., Wang L. (2021). Promoting the Thermoelectric Performance of Single-Walled Carbon Nanotubes by Inserting Discotic Liquid-Crystal Molecules. ACS Sustain. Chem. Eng..

[78] Davies J.H. (1997). The Physics of Low-Dimensional Semiconductors: An Introduction.

[79] Liu Y., Guo J., Zhu E., Liao L., Lee S.J., Ding M., Shakir I., Gambin V., Huang Y., Duan X. (2018). Approaching the Schottky-Mott Limit in van Der Waals Metal-Semiconductor Junctions. Nature.

[80] Vashaee D., Shakouri A. (2004). Improved Thermoelectric Power Factor in Metal-Based Superlattices. Phys. Rev. Lett..

[81] Liu M., Qin X.Y. (2012). Enhanced Thermoelectric Performance through Energy-Filtering Effects in Nanocomposites Dispersed with Metallic Particles. Appl. Phys. Lett..

[82] Heremans J.P., Thrush C.M., Morelli D.T. (2005). Thermopower Enhancement in PbTe with Pb Precipitates. J. Appl. Phys..

[83] Ko D.K., Kang Y., Murray C.B. (2011). Enhanced Thermopower via Carrier Energy Filtering in Solution-Processable Pt-Sb_2_Te_3_ Nanocomposites. Nano Lett..

[84] Zhou Y., Zhang M.Y., Liang S. (2021). Improved Thermoelectric Performance of Cu_2_O-Cr/Sn Composite Powder. Chem. Phys. Lett..

[85] Gao L., Wang S., Liu R., Zha X., Sun N., Wang S., Wang J., Fu G. (2016). Enhanced Thermoelectric Performance of CdO: Ag Nanocomposites. Dalton Trans..

[86] Milnes A.G., Feucht D.L. (1972). Heterojunctions and Metal Semiconductor Junctions.

[87] Mayergoyz I.D. (1986). Solution of the Nonlinear Poisson Equation of Semiconductor Device Theory. J. Appl. Phys..

[88] Wu H.J., Carrete J., Zhang Z.Y., Qu Y.Q., Shen X.T., Wang Z., Zhao L.D., He J.Q. (2014). Strong Enhancement of Phonon Scattering through Nanoscale Grains in Lead Sulfide Thermoelectrics. NPG Asia Mater..

[89] Seto J.Y.W. (1975). The Electrical Properties of Polycrystalline Silicon Films. J. Appl. Phys..

[90] Taylor W.E., Odell N.H., Fan H.Y. (1952). Grain Boundary Barriers in Germanium. Phys. Rev..

[91] Zhou Z.W., Yang J.Y., Jiang Q.H., Zhang D., Xin J.W., Li X., Ren Y.Y., He X. (2017). Thermoelectric Performance of SnTe with ZnO Carrier Compensation, Energy Filtering, and Multiscale Phonon Scattering. J. Am. Ceram. Soc..

[92] Dou Y.C., Qin X.Y., Li D., Li L.L., Zou T.H., Wang Q.Q. (2013). Enhanced Thermopower and Thermoelectric Performance through Energy Filtering of Carriers in (Bi_2_Te_3_)_(0.2)_(Sb_2_Te_3_)_(0.8)_ Bulk Alloy Embedded with Amorphous SiO_2_ Nanoparticles. J. Appl. Phys..

[93] Fang T., Li X., Hu C., Zhang Q., Yang J., Zhang W., Zhao X., Singh D.J., Zhu T. (2019). Complex Band Structures and Lattice Dynamics of Bi_2_Te_3_-Based Compounds and Solid Solutions. Adv. Funct. Mater..

[94] Li Y., Dou Y., Qin X., Zhang J., Xin H., Li D., Song C., Zou T., Liu Y., Li C. (2016). Enhanced Thermoelectric Figure of Merit in P-Type β-Zn_4_Sb_3_/Bi_0.4_Sb_1.6_Te_3_ Nanocomposites. RSC Adv..

[95] Nguyen T.H., Enju J., Ono T. (2019). Enhancement of Thermoelectric Properties of Bismuth Telluride Composite with Gold Nano-Particles Inclusions Using Electrochemical Co-Deposition. J. Electrochem. Soc..

[96] Madavali B., Kim H.S., Lee K.H., Hong S.J. (2017). Enhanced Seebeck Coefficient by Energy Filtering in Bi-Sb-Te Based Composites with Dispersed Y_2_O_3_ Nanoparticles. Intermetallics.

[97] Ravich I.I. (1970). Semiconducting Lead Chalcogenide.

[98] Zou T., Qin X., Zhang Y., Li X., Zeng Z., Li D., Zhang J., Xin H., Xie W., Weidenkaff A. (2015). Enhanced Thermoelectric Performance of β-Zn_4_Sb_3_ Based Nanocomposites through Combined Effects of Density of States Resonance and Carrier Energy Filtering. Sci. Rep..

[99] Xiong Z., Chen X., Zhao X., Bai S., Huang X., Chen L. (2009). Effects of Nano-TiO_2_ Dispersion on the Thermoelectric Properties Offilled-Skutterudite Ba_0.22_Co_4_Sb_12_. Solid State Sci..

[100] Dette C., Perez-Osorio M.A., Kley C.S., Punke P., Patrick C.E., Jacobson P., Giustino F., Jung S.J., Kern K. (2014). TiO_2_ Anatase with a Bandgap in the Visible Region. Nano Lett..

[101] Yang Y.-X., Wu Y.-H., Zhang Q., Cao G.-S., Zhu T.-J., Zhao X.-B. (2020). Enhanced Thermoelectric Performance of Bi_2_Se_3_/TiO_2_ Composite. Rare Met..

[102] Ou C., Hou J., Wei T.-R., Jiang B., Jiao S., Li J.-F., Zhu H. (2015). High Thermoelectric Performance of All-Oxide Heterostructures with Carrier Double-Barrier Filtering Effect. NPG Asia Mater..

[103] Yang X., Chen S., Zhang H., Lv F., Fan W., Wang W., Munir Z.A. (2018). Thermoelectric Properties and Transport Mechanism of Pure and Bi-Doped SiNWs-Mg_2_Si. Phys. Status Solidi A Appl. Mater. Sci..

[104] Hu Q., Wang K., Zhang Y., Li X., Song H. (2018). Enhanced Thermoelectric Properties of Nano SiC Dispersed Bi_2_Sr_2_Co_2_Oy Ceramics. Mater. Res. Express.

[105] Zhou Z., Li J., Fan Y., Zhang Q., Lu X., Fan S., Kikuchi K., Nomura N., Kawasaki A., Wang L. (2019). Uniform Dispersion of SiC in Yb-Filled Skutterudite Nanocomposites with High Thermoelectric and Mechanical Performance. Scr. Mater..

[106] Zhang W., Zhu K., Liu J., Wang J., Yan K., Liu P., Wang Y. (2019). Enhanced Thermoelectric Properties of Nano-SiC Dispersed NaCo_2_O_4_ Composites. Funct. Mater. Lett..

[107] Xia Y., Park J., Zhou F., Ozoliņš V. (2019). High Thermoelectric Power Factor in Intermetallic CoSi Arising from Energy Filtering of Electrons by Phonon Scattering. Phys. Rev. Appl..

[108] Ahmad S., Basu R., Sarkar P., Singh A., Bohra A., Bhattacharya S., Bhatt R., Meshram K.N., Samanta S., Bhatt P. (2018). Enhanced Thermoelectric Figure-of-Merit of p-Type SiGe through TiO_2_ Nanoinclusions and Modulation Doping of Boron. Materialia.

[109] Felizco J.C., Uenuma M., Fujii M.N., Uraoka Y. (2021). Improved Thermoelectric Power Factor of InGaZnO/SiO Thin Film Transistor via Gate-Tunable Energy Filtering. IEEE Electron Device Lett..

[110] Solá F., Dynys F.W. (2015). Probing the Mechanical Properties and Microstructure of WSi2/SixGe1−x Multiphase Thermoelectric Material by Nanoindentation, Electron and Focused Ion Beam Microscopy Methods. J. Alloy. Compd..

[111] Neophytou N., Zianni X., Kosina H., Frabboni S., Lorenzi B., Narducci D. (2013). Simultaneous Increase in Electrical Conductivity and Seebeck Coefficient in Highly Boron-Doped Nanocrystalline Si. Nanotechnology.

[112] Kang H.B., Poudel B., Li W., Lee H., Saparamadu U., Nozariasbmarz A., Kang M.G., Gupta A., Heremans J.J., Priya S. (2020). Decoupled Phononic-Electronic Transport in Multi-Phase n-Type Half-Heusler Nanocomposites Enabling Efficient High Temperature Power Generation. Mater. Today.

[113] Pakdel A., Guo Q., Nicolosi V., Mori T. (2018). Enhanced Thermoelectric Performance of Bi-Sb-Te/Sb_2_O_3_ Nanocomposites by Energy Filtering Effect. J. Mater. Chem. A.

[114] Li S., Liu Y., Liu F., He D., He J., Luo J., Xiao Y., Pan F. (2018). Effective Atomic Interface Engineering in Bi_2_Te_2.7_Se_0.3_ Thermoelectric Material by Atomic-Layer-Deposition Approach. Nano Energy.

[115] Güneş E., Wickleder M.S., Müller E., Elm M.T., Klar P.J. (2018). Improved Thermoelectric Properties of Nanostructured Composites out of Bi_1ࢤx_Sb_x_ Nanoparticles and Carbon Phases. AIP Adv..

[116] Wang Y.S., Huang L.L., Li D., Zhang J., Qin X.Y. (2018). Enhanced Thermoelectric Performance of Bi_0.4_Sb_1.6_Te_3_ Based Composites with CuInTe_2_ Inclusions. J. Alloys Compd..

[117] Cao R., Zhu Z., Li X.-J., Hu X., Song H. (2019). Enhanced Thermoelectric Properties of the Lu-Doped and CNT-Dispersed Bi_2_Te_3_ Alloy. Appl. Phys. A Mater. Sci. Process..

[118] Wan X., Liu Z., Sun L., Jiang P., Bao X. (2020). Synergetic Enhancement of Thermoelectric Performance in a Bi_0.5_Sb_1.5_Te_3_/SrTiO_3_ Heterostructure. J. Mater. Chem. A.

[119] Park N.-W., Lee W.-Y., Yoon Y.-S., Kim G.-S., Yoon Y.-G., Lee S.-K. (2019). Achieving Out-of-Plane Thermoelectric Figure of Merit ZT = 1.44 in a p-Type Bi_2_Te_3_/Bi_0.5_Sb_1.5_Te_3_ Superlattice Film with Low Interfacial Resistance. ACS Appl. Mater. Interfaces.

[120] Li Y., Wang X., Liu G., Shin B., Shan F. (2019). High Thermoelectric Efficiency of P-Type BiSbTe-Based Composites with CuGaTe_2_ Nanoinclusions. Scr. Mater..

[121] Lwin M.L., Dharmaiah P., Min B.H., Song G., Jung K.Y., Hong S.-J. (2021). Tuning of Thermoelectric Transport Properties via the Formation of Hierarchical Structures in Bi-Doped Gd_2_O_3_/Bi_0.5_Sb_1.5_Te_3_ Nanocomposites. Int. J. Energy Res..

[122] Ahmad M., Kodan N., Ghosh A., Mehta B.R. (2020). The Nature of 2D:3D SnS:Bi_2_Te_3_ Interface and Its Effect on Enhanced Electrical and Thermoelectric Properties. J. Alloy. Compd..

[123] Zhao L., Qiu W., Sun Y., Chen L., Deng H., Yang L., Shi X., Tang J. (2021). Enhanced Thermoelectric Performance of Bi_0.3_Sb_1.7_Te_3_ Based Alloys by Dispersing TiC Ceramic Nanoparticles. J. Alloy. Compd..

[124] Zhang Y., Xing C., Liu Y., Li M., Xiao K., Guardia P., Lee S., Han X., Ostovari Moghaddam A., Josep Roa J. (2021). Influence of Copper Telluride Nanodomains on the Transport Properties of N-Type Bismuth Telluride. Chem. Eng. J..

[125] Hwang J., Lee M., Yu B.-K., Han M.-K., Kim W., Kim J., Al Rahal Al Orabi R., Wang H., Acharya S., Kim J. (2021). Enhancement of Thermoelectric Performance in a Non-Toxic CuInTe_2_/SnTe Coated Grain Nanocomposite. J. Mater. Chem. A.

[126] Dharmaiah P., Nagarjuna C., Sharief P., Hong S.-J. (2021). Synergetic Effects of Co-Dispersed Cu and Insulating HfO_2_ Nanoparticles Enabled High Thermoelectric Figure of Merit in Bi_0.5_Sb_1.5_Te_3_ Composites. Appl. Surf. Sci..

[127] Pei Y.L., Wu H., Wu D., Zheng F., He J. (2014). High Thermoelectric Performance Realized in a BiCuSeO System by Improving Carrier Mobility through 3D Modulation Doping. J. Am. Chem. Soc..

[128] Dingle R., Störmer H.L., Gossard A.C., Wiegmann W. (1978). Electron Mobilities in Modulation-doped Semiconductor Heterojunction Superlattices. Appl. Phys. Lett..

[129] Mimura T., Hiyamizu S., Fujii T., Nanbu K. (1980). A New Field-Effect Transistor with Selectively Doped GaAs/n-Al_x_Ga_1−x_As Heterojunctions. Jpn. J. Appl. Phys..

[130] Pfeiffer L., West K.W., Stormer H.L., Baldwin K.W. (1998). Electron Mobilities Exceeding 10^7^ cm^2^/V s in Modulation-doped GaAs. Appl. Phys. Lett..

[131] Yamini S.A., Mitchell D.R.G., Gibbs Z.M., Santos R., Patterson V., Li S., Pei Y.Z., Dou S.X., Snyder G.J. (2015). Heterogeneous Distribution of Sodium for High Thermoelectric Performance of P-Type Multiphase Lead-Chalcogenides. Adv. Energy Mater..

[132] Tian Y., Sakr M.R., Kinder J.M., Liang D., MacDonald M.J., Qiu R.L.J., Gao H.-J., Gao X.P.A. (2012). One-Dimensional Quantum Confinement Effect Modulated Thermoelectric Properties in InAs Nanowires. Nano Lett..

[133] Moon J., Kim J.-H., Chen Z.C.Y., Xiang J., Chen R. (2013). Gate-Modulated Thermoelectric Power Factor of Hole Gas in Ge-Si Core-Shell Nanowires. Nano Lett..

[134] Liang W., Hochbaum A.I., Fardy M., Rabin O., Zhang M., Yang P. (2009). Field-Effect Modulation of Seebeck Coefficient in Single PbSe Nanowires. Nano Lett..

[135] Curtin B.M., Codecido E.A., Krämer S., Bowers J.E. (2013). Field-Effect Modulation of Thermoelectric Properties in Multigated Silicon Nanowires. Nano Lett..

[136] Neophytou N., Thesberg M. (2016). Modulation Doping and Energy Filtering as Effective Ways to Improve the Thermoelectric Power Factor. J. Comput. Electron..

[137] Zhang Y., Brorsson J., Qiu R., Palmqvist A.E.C. (2021). Enhanced Thermoelectric Performance of Ba_8_Ga_16_Ge_30_ Clathrate by Modulation Doping and Improved Carrier Mobility. Adv. Electron. Mater..

[138] Rathore E., Guin S.N., Biswas K. (2020). Enhancement of Thermoelectric Performance of N-Type AgBi_1+x_Se_2_ via Improvement of the Carrier Mobility by Modulation Doping. Bull. Mater. Sci..

[139] Peng Y., Lai H., Liu C., Gao J., Kurosawa M., Nakatsuka O., Takeuchi T., Zaima S., Tanemura S., Miao L. (2020). Realizing High Thermoelectric Performance in P-Type Si_1−x_-YGexSny Thin Films at Ambient Temperature by Sn Modulation Doping. Appl. Phys. Lett..

[140] Souda D., Shimizu K., Ohishi Y., Muta H., Yagi T., Kurosaki K. (2020). High Thermoelectric Power Factor of Si–Mg_2_Si Nanocomposite Ribbons Synthesized by Melt Spinning. ACS Appl. Energy Mater..

[141] Lee D., Zhou J., Chen G., Shao-Horn Y. (2019). Enhanced Thermoelectric Properties for PEDOT:PSS/Undoped Ge Thin-Film Bilayered Heterostructures. Adv. Electron. Mater..

[142] Hui S., Gao W., Lu X., Panda A., Bailey T.P., Page A.A., Forrest S.R., Morelli D.T., Pan X., Pipe K.P. (2017). Engineering Temperature-Dependent Carrier Concentration in Bulk Composite Materials via Temperature-Dependent Fermi Level Offset. Adv. Energy Mater..

[143] Feng B., Li G., Pan Z., Hu X., Liu P., He Z., Li Y., Fan X. (2019). Enhanced Thermoelectric Properties in BiCuSeO Ceramics by Pb/Ni Dual Doping and 3D Modulation Doping. J. Solid State Chem..

[144] Feng B., Li G., Pan Z., Hu X., Liu P., Li Y., He Z., Fan X. (2019). Enhanced Thermoelectric Performances in BiCuSeO Oxyselenides via Er and 3D Modulation Doping. Ceram. Int..

[145] Feng B., Li G., Pan Z., Hu X., Liu P., He Z., Li Y., Fan X. (2018). Enhanced Thermoelectric Performance in BiCuSeO Oxyselenides via Ba/Te Dual-Site Substitution and 3D Modulation Doping. J. Solid State Chem..

[146] Wu D., Zhao L.-D., Tong X., Li W., Wu L., Tan Q., Pei Y., Huang L., Li J.-F., Zhu Y. (2015). Superior Thermoelectric Performance in PbTe–PbS Pseudo-Binary: Extremely Low Thermal Conductivity and Modulated Carrier Concentration. Energy Environ. Sci..

[147] Rowe D.M. (2006). Thermoelectrics Handbook.

[148] Hong M., Wang Y., Feng T., Sun Q., Xu S., Matsumura S., Pantelides S.T., Zou J., Chen Z.G. (2019). Strong Phonon-Phonon Interactions Securing Extraordinary Thermoelectric Ge1- XSb XTe with Zn-Alloying-Induced Band Alignment. J. Am. Chem. Soc..

[149] Bao D.Y., Chen J., Yu Y., Liu W.D., Huang L.S., Han G., Tang J., Zhou D.L., Yang L., Chen Z.G. (2020). Texture-Dependent Thermoelectric Properties of Nano-Structured Bi_2_Te_3_. Chem. Eng. J..

[150] Alam H., Ramakrishna S. (2013). A Review on the Enhancement of Figure of Merit from Bulk to Nano-Thermoelectric Materials. Nano Energy.

[151] Dresselhaus M.S., Chen G., Tang M.Y., Yang R.G., Lee H., Wang D.Z., Ren Z.F., Fleurial J.P., Gogna P. (2007). New Directions for Low-Dimensional Thermoelectric Materials. Adv. Mater..

[152] Chen X., Parker D., Singh D.J. (2013). Acoustic Impedance and Interface Phonon Scattering in Bi_2_Te_3_ and Other Semiconducting Materials. Phys. Rev. B.

[153] Yang J., Meisner G.P., Chen L. (2004). Strain Field Fluctuation Effects on Lattice Thermal Conductivity of ZrNiSn-Based Thermoelectric Compounds. Appl. Phys. Lett..

[154] Yan X., Liu W.S., Wang H., Chen S., Shiomi J., Esfarjani K., Wang H.Z., Wang D.Z., Chen G., Ren Z.F. (2012). Stronger Phonon Scattering by Larger Differences in Atomic Mass and Size in P-Type Half-Heuslers Hf1-XTixCoSb_0.8_Sn_0.2_. Energy Environ. Sci..

[155] Xie H., Wang H., Fu C., Liu Y., Snyder G.J., Zhao X., Zhu T. (2014). The Intrinsic Disorder Related Alloy Scattering in ZrNiSn Half-Heusler Thermoelectric Materials. Sci. Rep..

[156] Bhattacharya S., Skove M.J., Russell M., Tritt T.M., Xia Y., Ponnambalam V., Poon S.J., Thadhani N. (2008). Effect of Boundary Scattering on the Thermal Conductivity of TiNiSn-Based Half-Heusler Alloys. Phys. Rev. B.

[157] Bhattacharya S., Tritt T.M., Xia Y., Ponnambalam V., Poon S.J., Thadhani N. (2002). Grain Structure Effects on the Lattice Thermal Conductivity of Ti-Based Half-Heusler Alloys. Appl. Phys. Lett..

[158] Yan X., Joshi G., Liu W., Lan Y., Wang H., Lee S., Simonson J.W., Poon S.J., Tritt T.M., Chen G. (2011). Enhanced Thermoelectric Figure of Merit of P-Type Half-Heuslers. Nano Lett..

[159] Joshi G., Yan X., Wang H., Liu W., Chen G., Ren Z. (2011). Enhancement in Thermoelectric Figure-Of-Merit of an N-Type Half-Heusler Compound by the Nanocomposite Approach. Adv. Energy Mater..

[160] Jiang P., Lindsay L., Huang X., Koh Y.K. (2018). Interfacial Phonon Scattering and Transmission Loss in >1 Μm Thick Silicon-on-Insulator Thin Films. Phys. Rev. B.

[161] Klemens P.G., Simon F.E. (1951). The Thermal Conductivity of Dielectric Solids at Low Temperatures (Theoretical). Proc. R. Soc. Lond. Ser. A Math. Phys. Sci..

[162] Holland M.G. (1963). Analysis of Lattice Thermal Conductivity. Phys. Rev..

[163] Callaway J. (1959). Model for Lattice Thermal Conductivity at Low Temperatures. Phys. Rev..

[164] Limarga A.M., Shian S., Leckie R.M., Levi C.G., Clarke D.R. (2014). Thermal Conductivity of Single- and Multi-Phase Compositions in the ZrO_2_-Y_2_O_3_-Ta_2_O_5_ System. J. Eur. Ceram. Soc..

[165] Zhao H.Z., Pokheral M., Zhu G.H., Chen S., Lukas K., Jie Q., Opeil C., Chen G., Ren Z.F. (2011). Dramatic Thermal Conductivity Reduction by Nanostructures for Large Increase in Thermoelectric Figure-of-Merit of FeSb_2_. Appl. Phys. Lett..

[166] Gurunathan R., Hanus R., Jeffrey Snyder G. (2020). Alloy Scattering of Phonons. Mater. Horiz..

[167] Nan C.-W., Birringer R. (1998). Determining the Kapitza Resistance and the Thermal Conductivity of Polycrystals: A Simple Model. Phys. Rev. B.

[168] Kinsler L.E., Frey A.R., Coppens A.B., Sanders J.V. (1999). Fundamentals of Acoustics.

[169] James D., Lu X., Nguyen A.C., Morelli D., Brock S.L. (2015). Design of Lead Telluride Based Thermoelectric Materials through Incorporation of Lead Sulfide Inclusions or Ligand Stripping of Nanosized Building Blocks. J. Phys. Chem. C.

[170] Ahmad K., Almutairi Z., Wan C. (2020). Thermoelectric Properties of PbTe-Based Graphene Nanocomposite. J. Mater. Sci. Mater. Electron..

[171] Aminorroaya Yamini S., Wang H., Gibbs Z.M., Pei Y., Mitchell D.R.G., Dou S.X., Snyder G.J. (2014). Thermoelectric Performance of Tellurium-Reduced Quaternary p-Type Lead–Chalcogenide Composites. Acta Mater..

[172] Falkenbach O., Hartung D., Klar P.J., Koch G., Schlecht S. (2013). Thermoelectric Properties of Nanostructured Bismuth-Doped Lead Telluride Bi_x_(PbTe)_1−x_ Prepared by Co-Ball-Milling. J. Electron. Mater..

[173] Falkenbach O., Schmitz A., Hartung D., Dankwort T., Koch G., Kienle L., Klar P.J., Mueller E., Schlecht S. (2016). Effect of Preparation Procedure and Nanostructuring on the Thermoelectric Properties of the Lead Telluride-Based Material System AgPb_m_BiTe_2+m_ (BLST-m). J. Appl. Phys..

[174] Keshavarz M.K., Vasilevskiy D., Masut R.A., Turenne S. (2014). Synthesis and Characterization of Bismuth Telluride-Based Thermoelectric Nanocomposites Containing MoS_2_ Nano-Inclusions. Mater. Charact..

[175] Trawiński B., Bochentyn B., Gostkowska N., Łapiński M., Miruszewski T., Kusz B. (2018). Structure and Thermoelectric Properties of Bismuth Telluride—Carbon Composites. Mater. Res. Bull..

[176] Yang G., Sang L., Yun F.F., Mitchell D.R.G., Casillas G., Ye N., See K., Pei J., Wang X., Li J. (2021). Significant Enhancement of Thermoelectric Figure of Merit in BiSbTe-Based Composites by Incorporating Carbon Microfiber. Adv. Funct. Mater..

[177] Zhang J., Wu D., He D., Feng D., Yin M., Qin X., He J. (2017). Extraordinary Thermoelectric Performance Realized in N-Type PbTe through Multiphase Nanostructure Engineering. Adv. Mater..

[178] Sootsman J.R., He J.Q., Dravid V.P., Li C.P., Uher C., Kanatzidis M.G. (2009). High Thermoelectric Figure of Merit and Improved Mechanical Properties in Melt Quenched PbTe-Ge and PbTe-Ge_1−x_Si_x_ Eutectic and Hypereutectic Composites. J. Appl. Phys..

[179] Gaultois M.W., Sparks T.D., Borg C.K.H., Seshadri R., Bonificio W.D., Clarke D.R. (2013). Data-Driven Review of Thermoelectric Materials: Performance and Resource Considerations. Chem. Mater..

[180] Gaultois M.W., Sparks T.D., Borg C.K.H., Seshadri R., Bonificio W.D., Clarke D.R. Energy Materials Datamining. http://www.mrl.ucsb.edu:8080/datamine/about.jsp.

[181] Kittel C. (2018). Introduction to Solid State Physics.

[182] Chung D.Y., Hogan T., Brazis P., Rocci-Lane M., Kannewurf C., Bastea M., Uher C., Kanatzidis M.G. (2000). CsBi_4_Te_6_: A High-Performance Thermoelectric Material for Low-Temperature Applications. Science.

[183] Chung D.-Y., Choi K.-S., Iordanidis L., Schindler J.L., Brazis P.W., Kannewurf C.R., Chen B., Hu S., Uher C., Kanatzidis M.G. (1997). High Thermopower and Low Thermal Conductivity in Semiconducting Ternary K−Bi−Se Compounds. Synthesis and Properties of β-K_2_Bi_8_Se_13_ and K_2.5_Bi_8.5_Se_14_ and Their Sb Analogues. Chem. Mater..

[184] Gascoin F., Maignan A. (2011). Order-Disorder Transition in AgCrSe_2_: A New Route to Efficient Thermoelectrics. Chem. Mater..

[185] Hsu K.F., Loo S., Guo F., Chen W., Dyck J.S., Uher C., Hogan T., Polychroniadis E.K., Kanatzidis M.G. (2004). Cubic AgPb(_m_)SbTe(_2+m_): Bulk Thermoelectric Materials with High Figure of Merit. Science.

[186] Jungwirth T., Wunderlich J., Olejnik K. (2012). Spin Hall Effect Devices. Nat. Mater..

[187] Kanatzidis M.G., McCarthy T.J., Tanzer T.A., Chen L.-H., Iordanidis L., Hogan T., Kannewurf C.R., Uher C., Chen B. (1996). Synthesis and Thermoelectric Properties of the New Ternary Bismuth Sulfides KBi_6.33_S_10_ and K_2_Bi_8_S_13_. Chem. Mater..

[188] Kurosaki K., Kosuga A., Yamanaka S. (2003). Thermoelectric Properties of Chevrel Phase Mo6Te8-XSx. J. Alloys Compd..

[189] Kurosaki K., Kosuga A., Muta H., Uno M., Yamanaka S. (2005). Ag_9_TlTe_5_: A High-Performance Thermoelectric Bulk Material with Extremely Low Thermal Conductivity. Appl. Phys. Lett..

[190] Larouche S., Tsai Y.J., Tyler T., Jokerst N.M., Smith D.R. (2012). Infrared Metamaterial Phase Holograms. Nat. Mater..

[191] Liu H., Shi X., Xu F., Zhang L., Zhang W., Chen L., Li Q., Uher C., Day T., Snyder G.J. (2012). Copper Ion Liquid-like Thermoelectrics. Nat. Mater..

[192] Martin C.D., Costa A., Dering B., Hoshino N., Wu Y.J., Thierry G. (2012). Effects of Speed of Word Processing on Semantic Access: The Case of Bilingualism. Brain Lang..

[193] May A.F., Flage-Larsen E., Snyder G.J. (2010). Electron and Phonon Scattering in the High-Temperature Thermoelectric La_3_Te_4−z_M_z_ (M = Sb,Bi). Phys. Rev. B.

[194] McGuire M.A., Reynolds T.K., DiSalvo F.J. (2005). Exploring Thallium Compounds as Thermoelectric Materials: Seventeen New Thallium Chalcogenides. Chem. Mater..

[195] Ohta M., Yamamoto A., Obara H. (2010). Thermoelectric Properties of Chevrel-Phase Sulfides M_x_ Mo_6_S_8_ (M: Cr, Mn, Fe, Ni). J. Electron. Mater..

[196] Orava J., Greer A.L., Gholipour B., Hewak D.W., Smith C.E. (2012). Characterization of Supercooled Liquid Ge2Sb2Te5 and Its Crystallization by Ultrafast-Heating Calorimetry. Nat. Mater..

[197] Pei Y., Shi X., LaLonde A., Wang H., Chen L., Snyder G.J. (2011). Convergence of Electronic Bands for High Performance Bulk Thermoelectrics. Nature.

[198] Rogers E.T., Lindberg J., Roy T., Savo S., Chad J.E., Dennis M.R., Zheludev N.I. (2012). A Super-Oscillatory Lens Optical Microscope for Subwavelength Imaging. Nat. Mater..

[199] Scherrer S., Scherrer H., Rowe D. (1995). Bismuth Telluride, Antimony Telluride, and Their Solid Solutions. CRC Handbook of Thermoelectrics.

[200] Sharp J.W., Sales B.C., Mandrus D.G., Chakoumakos B.C. (1999). Thermoelectric Properties of Tl_2_SnTe_5_ and Tl_2_GeTe_5_. Appl. Phys. Lett..

[201] Skrabek E., Trimmer D., Rowe D. (1995). Properties of the General TAGS System. CRC Handbook of Thermoelectrics.

[202] Wan C., Wang Y., Wang N., Koumoto K. (2010). Low-Thermal-Conductivity (MS)_1+x_(TiS_2_)_2_ (M = Pb, Bi, Sn) Misfit Layer Compounds for Bulk Thermoelectric Materials. Materials.

[203] Wang W.H. (2012). Metallic Glasses: Family Traits. Nat. Mater..

[204] Warren S.C., Perkins M.R., Adams A.M., Kamperman M., Burns A.A., Arora H., Herz E., Suteewong T., Sai H., Li Z. (2012). A Silica Sol-Gel Design Strategy for Nanostructured Metallic Materials. Nat. Mater..

[205] Cui J.L., Xue H.F., Xiu W.J. (2007). Preparation and Thermoelectric Properties of P-Type (Ga_2_Te_3_)x–(Bi_0.5_Sb_1.5_Te_3_)_1−x_ (X = 0–0.2) Alloys Prepared by Spark Plasma Sintering. Intermetallics.

[206] Gayner C., Nandihalli N. (2020). Enhancement of Thermoelectric Performance of PbTe by Embedding NaCl. Materialia.

[207] Chang C.-C., Liu C.-H., Wu C.-C., Bag P., Kuo Y.-K. (2020). Thermoelectric Properties of (HgTe)_0.55_(PbTe)_0.45_ Eutectic Composite with In Doping. Mater. Res. Bull..

[208] Nandihalli N., Pai Y.-H., Liu C.-J. (2020). Thermoelectric Properties of Pb_0.833_Na_0.017_(Zn_0.85_Al_0.15_)_0.15_Te-Te Composite. Ceram. Int..

[209] Zhu C., Zhang J., Ming H., Lou X., Huang L., Chen T., Zhang B., Li D., Xin H., Qin X. (2020). Enhanced Thermoelectric Performance of PbTe Based Materials by Bi Doping and Introducing MgO Nanoparticles. Appl. Phys. Lett..

[210] Yang Z.-R., Liu C.-J. (2020). Thermoelectric Transport in P-Type (Pb_0.98_Na_0.02_Te)_1−x_(Zn_0.85_Al_0.15_Te)_x_-Te Composites Fabricated Using a Combination of Hydrothermal Synthesis and Evacuating-and-Encapsulating Sintering. J. Electron. Mater..

[211] Zhu T., Xie H., Zhang C., Cheng X., Zhang J., Poudeu P.F.P., Tan G., Yan Y., Liu W., Su X. (2019). Enhanced Mechanical Properties of Na_0.02_Pb_0.98_Te/MoTe_2_ Thermoelectric Composites Through in-Situ-Formed MoTe_2_. ACS Appl. Mater. Interfaces.

[212] Gao J., Mao T., Lv T., Li Z., Xu G. (2018). Thermoelectric Performance of N-Type (PbTe)_1−x_(CoTe)_x_ Composite Prepared by High Pressure Sintering Method. J. Mater. Sci. Mater. Electron..

[213] Ginting D., Lin C.-C., Rathnam L., Hwang J., Kim W., Al Orabi R.A.r., Rhyee J.-S. (2017). Dataset on the Electronic and Thermal Transport Properties of Quaternary Compounds of (PbTe)_0.95−x_(PbSe)_x_(PbS)_0.05_. Data Brief.

[214] Rösch A.G., Giunta F., Mallick M.M., Franke L., Gall A., Aghassi-Hagmann J., Schmalian J., Lemmer U. (2021). Improved Electrical, Thermal, and Thermoelectric Properties Through Sample-to-Sample Fluctuations in Near-Percolation Threshold Composite Materials. Adv. Simul..

[215] Bruggeman D.A.G. (1935). Berechnung Verschiedener Physikalischer Konstanten von Heterogenen Substanzen. I. Dielektrizitätskonstanten Und Leitfähigkeiten Der Mischkörper Aus Isotropen Substanzen. Ann. Physik.

[216] Landauer R. (1952). The Electrical Resistance of Binary Metallic Mixtures. J. Appl. Phys..

[217] Vaney J.B., Piarristeguy A., Ohorodniichuck V., Ferry O., Pradel A., Alleno E., Monnier J., Lopes E.B., Goncalves A.P., Delaizir G. (2015). Effective Medium Theory Based Modeling of the Thermoelectric Properties of Composites: Comparison between Predictions and Experiments in the Glass-Crystal Composite System Si_10_As_15_Te_75_-Bi_0.4_Sb_1.6_Te_3_. J. Mater. Chem. C.

[218] Sonntag J. (2016). Comment on Effective Medium Theory Based Modeling of the Thermoelectric Properties of Composites: Comparison between Predictions and Experiments in the Glass–Crystal Composite System Si_10_As_15_Te_75_-Bi_0.4_Sb_1.6_Te_3_ by J.-B. Vaney et al., J. Mater. Chem. C, **2015**, *3*, 11090. J. Mater. Chem. C.

[219] Angst S., Wolf D.E. (2016). Network Theory for Inhomogeneous Thermoelectrics. New J. Phys..

[220] Gather F., Heiliger C., Klar P.J. (2011). NeMo: A Network Model Program for Analyzing the Thermoelectric Properties of Meso and Nanostructured Composite Materials. Prog. Solid State Chem..

[221] Aboudi J., Haj-Ali R. (2016). A Fully Coupled Thermal–Electrical–Mechanical Micromodel for Multi-Phase Periodic Thermoelectrical Composite Materials and Devices. Int. J. Solids Struct..

[222] Monticelli A. (1999). State Estimation in Electric Power Systems: A Generalized Approach.

[223] Razavi B. (2013). Fundamentals of Microelectronics.

[224] Roche S. (2010). Graphene Gets a Better Gap. Nat. Nanotechnol..

[225] Suh D., Lee S., Mun H., Park S.H., Lee K.H., Kim S.W., Choi J.Y., Baik S. (2015). Enhanced Thermoelectric Performance of Bi0.5Sb1.5Te3-Expanded Graphene Composites by Simultaneous Modulation of Electronic and Thermal Carrier Transport. Nano Energy.

[226] Yadav S., Chaudhary S., Pandya D.K. (2018). Effect of 2D MoS_2_ and Graphene Interfaces with CoSb_3_ Nanoparticles in Enhancing Thermoelectric Properties of 2D MoS_2_-CoSb_3_ and Graphene-CoSb_3_ Nanocomposites. Ceram. Int..

[227] Zhang Y., Ma H., Sun B., Liu B., Liu H., Kong L., Liu B., Jia X., Chen X. (2017). Thermoelectric Performance of Graphene Composited BiSbTe Bulks by High Pressure Synthesis. J. Alloys Compd..

[228] He Y., Zhang T.S., Shi X., Wei S.H., Chen L.D. (2015). High Thermoelectric Performance in Copper Telluride. NPG Asia Mater..

[229] Sottmann J., Valset K., Karlsen O.B., Tafto J. (2013). Synthesis and Measurement of the Thermoelectric Properties of Multiphase Composites: ZnSb Matrix with Zn_4_Sb_3_, Zn_3_P_2_, and Cu_5_Zn_8_. J. Electron. Mater..

[230] Yamini S.A., Brewis M., Byrnes J., Santos R., Manettas A., Pei Y.Z. (2015). Fabrication of Thermoelectric Materials—Thermal Stability and Repeatability of Achieved Efficiencies. J. Mater. Chem. C.

[231] Aminorroaya Yamini S., Mitchell D.R.G., Wang H., Gibbs Z.M., Pei Y., Dou S.X., Snyder G.J. (2015). Origin of Resistivity Anomaly in P-Type Leads Chalcogenide Multiphase Compounds. AIP Adv..

[232] Shen J.J., Hu L.P., Zhu T.J., Zhao X.B. (2011). The Texture Related Anisotropy of Thermoelectric Properties in Bismuth Telluride Based Polycrystalline Alloys. Appl. Phys. Lett..

[233] Zhao X.B., Ji X.H., Zhang Y.H., Zhu T.J., Tu J.P., Zhang X.B. (2005). Bismuth Telluride Nanotubes and the Effects on the Thermoelectric Properties of Nanotube-Containing Nanocomposites. Appl. Phys. Lett..

[234] Bailyn M. (1962). Maximum Variational Principle for Conduction Problems in a Magnetic Field, and the Theory of Magnon Drag. Phys. Rev..

[235] Hirohata A., Yamada K., Nakatani Y., Prejbeanu I.L., Dieny B., Pirro P., Hillebrands B. (2020). Review on Spintronics: Principles and Device Applications. J. Magn. Magn. Mater..

[236] Costache M.V., Bridoux G., Neumann I., Valenzuela S.O. (2011). Magnon-Drag Thermopile. Nat. Mater..

[237] Watzman S.J., Duine R.A., Tserkovnyak Y., Boona S.R., Jin H., Prakash A., Zheng Y.H., Heremans J.P. (2016). Magnon-Drag Thermopower and Nernst Coefficient in Fe, Co, and Ni. Phys. Rev. B.

[238] Saito T., Nishio-Hamane D. (2021). Magnetic and Thermoelectric Properties of Co_2_MnT (T = Ga, Si) Heusler Compounds. Phys. B Condens. Matter.

[239] Saito T., Kamishima S. (2019). Magnetic and Thermoelectric Properties of Fe–Ti–Sn Alloys. IEEE Trans. Magn..

[240] Vikram F., Johnson D.D., Alam A. (2018). Enhanced Thermoelectric Performance of Mg_2_Si_1−x_Sn_x_ Codoped with Bi and Cr. Phys. Rev. B.

[241] Solomon G., Song E., Gayner C., Martinez J.A., Amouyal Y. (2021). Effects of Microstructure and Neodymium Doping on Bi_2_Te_3_ Nanostructures: Implications for Thermoelectric Performance. ACS Appl. Nano Mater..

[242] Jena A., Lee S.-C., Bhattacharjee S. (2021). Tuning the Lattice Thermal Conductivity in Bismuth Telluride via Cr Alloying. Phys. Rev. Appl..

[243] Das S., Valiyaveettil S.M., Chen K.-H., Suwas S., Mallik R.C. (2019). Thermoelectric Properties of Mn Doped BiCuSeO. Mater. Res. Express.

[244] Diakhate M.S., Hermann R.P., Mochel A., Sergueev I., Sondergaard M., Christensen M., Verstraete M.J. (2011). Thermodynamic, Thermoelectric, and Magnetic Properties of FeSb_2_: A Combined First-Principles and Experimental Study. Phys. Rev. B.

[245] Sun P., Oeschler N., Johnsen S., Iversen B.B., Steglich F. (2010). Narrow Band Gap and Enhanced Thermoelectricity in FeSb_2_. Dalton Trans..

[246] Franzen H., Sterner C. (1978). The X-Ray Photoelectron Spectra of MnS, MnSe, and MnTe. J. Solid State Chem..

[247] Podgorny M., Oleszkiewicz J. (1983). Electronic Structure of Antiferromagnetic MnTe. J. Phys. C Solid State Phys..

[248] Wasscher J.D., Haas C. (1964). Contribution of Magnon-Drag to the Thermoelectric Power of Antiferromagnetic Mn Te. Phys. Lett..

[249] Peng C., Zhang G., Wang C., Yan Y., Zheng H., Wang Y., Hu M. (2018). Improvement of Thermoelectricity Through Magnetic Interactions in Layered Cr_2_Ge_2_Te_6_. Phys. Status Solidi RRL Rapid Res. Lett..

[250] Kirkham M.J., dos Santos A.M., Rawn C.J., Lara-Curzio E., Sharp J.W., Thompson A.J. (2012). Abinitio Determination of Crystal Structures of the Thermoelectric Material MgAgSb. Phys. Rev. B.

[251] Zheng L., Li J., Zhou B., Liu H., Bu Z., Chen B., Ang R., Li W. (2019). Thermoelectric Properties of P-Type MnSe. J. Alloys Compd..

[252] Shimizu S., Shiogai J., Takemori N., Sakai S., Ikeda H., Arita R., Nojima T., Tsukazaki A., Iwasa Y. (2019). Giant Thermoelectric Power Factor in Ultrathin FeSe Superconductor. Nat. Commun..

[253] Takahashi H., Okazaki R., Ishiwata S., Taniguchi H., Okutani A., Hagiwara M., Terasaki I. (2016). Colossal Seebeck Effect Enhanced by Quasi-Ballistic Phonons Dragging Massive Electrons in FeSb_2_. Nat. Commun..

[254] Tsujii N., Mori T. (2013). High Thermoelectric Power Factor in a Carrier-Doped Magnetic Semiconductor CuFeS_2_. Appl. Phys. Express.

[255] Ang R., Khan A.U., Tsujii N., Takai K., Nakamura R., Mori T. (2015). Thermoelectricity Generation and Electron-Magnon Scattering in a Natural Chalcopyrite Mineral from a Deep-Sea Hydrothermal Vent. Angew. Chem. Int. Ed. Engl..

[256] Kikkawa T., Reitz D., Ito H., Makiuchi T., Sugimoto T., Tsunekawa K., Daimon S., Oyanagi K., Ramos R., Takahashi S. (2021). Observation of Nuclear-Spin Seebeck Effect. Nat. Commun..

[257] Uchida K., Takahashi S., Harii K., Ieda J., Koshibae W., Ando K., Maekawa S., Saitoh E. (2008). Observation of the Spin Seebeck Effect. Nature.

[258] Wang Y., Rogado N.S., Cava R.J., Ong N.P. (2003). Spin Entropy as the Likely Source of Enhanced Thermopower in Na(x)Co_2_O_4_. Nature.

[259] Bauer G.E., Saitoh E., van Wees B.J. (2012). Spin Caloritronics. Nat. Mater..

[260] Yu H.M., Brechet S.D., Ansermet J.P. (2017). Spin Caloritronics, Origin and Outlook. Phys. Lett. A.

[261] Erekhinsky M., Casanova F., Schuller I.K., Sharoni A. (2012). Spin-Dependent Seebeck Effect in Non-Local Spin Valve Devices. Appl. Phys. Lett..

[262] Marchal N., da Camara Santa Clara Gomes T., Abreu Araujo F., Piraux L. (2020). Large Spin-Dependent Thermoelectric Effects in NiFe-Based Interconnected Nanowire Networks. Nanoscale Res. Lett..

[263] Yamanoi K., Yafuso M., Miyazaki K., Kimura T. (2019). Signature of Spin-Dependent Seebeck Effect in Dynamical Spin Injection of Metallic Bilayer Structures. J. Phys. Mater..

[264] Ahmed F., Tsujii N., Mori T. (2017). Thermoelectric Properties of CuGa_1−x_Mn_x_Te_2_: Power Factor Enhancement by Incorporation of Magnetic Ions. J. Mater. Chem. A.

[265] Vaney J.B., Yamini S.A., Takaki H., Kobayashi K., Kobayashi N., Mori T. (2019). Magnetism-Mediated Thermoelectric Performance of the Cr-Doped Bismuth Telluride Tetradymite. Mater. Today Phys..

[266] Acharya S., Anwar S., Mori T., Soni A. (2018). Coupling of Charge Carriers with Magnetic Entropy for Power Factor Enhancement in Mn Doped Sn_1.03_Te for Thermoelectric Applications. J. Mater. Chem. C.

[267] Li W., Chen Z.W., Lin S.Q., Chang Y.J., Ge B.H., Chen Y., Pei Y.Z. (2015). Band and Scattering Tuning for High Performance Thermoelectric Sn_1−x_Mn_x_Te Alloys. J. Mater..

[268] He J., Tan X., Xu J., Liu G.-Q., Shao H., Fu Y., Wang X., Liu Z., Xu J., Jiang H. (2015). Valence Band Engineering and Thermoelectric Performance Optimization in SnTe by Mn-Alloying via a Zone-Melting Method. J. Mater. Chem. A.

[269] Graf T., Barth J., Blum C.G.F., Balke B., Felser C., Klaer P., Elmers H.-J. (2010). Phase-Separation-Induced Changes in the Magnetic and Transport Properties of the Quaternary Heusler Alloy Co_2_Mn_1−x_Ti_x_Sn. Phys. Rev. B.

[270] Liu Z., Zhu J., Wei P., Zhu W., Zhao W., Xia A., Xu D., Lei Y., Yu J. (2019). Candidate for Magnetic Doping Agent and High-Temperature Thermoelectric Performance Enhancer: Hard Magnetic M-Type BaFe_12_O_19_ Nanometer Suspension. ACS Appl. Mater. Interfaces.

[271] Zhao W., Liu Z., Wei P., Zhang Q., Zhu W., Su X., Tang X., Yang J., Liu Y., Shi J. (2017). Magnetoelectric Interaction and Transport Behaviours in Magnetic Nanocomposite Thermoelectric Materials. Nat. Nanotechnol..

[272] Lu R., Lopez J.S., Liu Y., Bailey T.P., Page A.A., Wang S., Uher C., Poudeu P.F.P. (2019). Coherent Magnetic Nanoinclusions Induce Charge Localization in Half-Heusler Alloys Leading to High-Tc Ferromagnetism and Enhanced Thermoelectric Performance. J. Mater. Chem. A.

[273] Vandendriessche S., Brullot W., Slavov D., Valev V.K., Verbiest T. (2013). Magneto-Optical Harmonic Susceptometry of Superparamagnetic Materials. Appl. Phys. Lett..

[274] Marghussian V. (2015). Magnetic Properties of Nano-Glass Ceramics. Nano-Glass Ceramics.

[275] Zhao W., Liu Z., Sun Z., Zhang Q., Wei P., Mu X., Zhou H., Li C., Ma S., He D. (2017). Superparamagnetic Enhancement of Thermoelectric Performance. Nature.

